# Modern and sub-fossil corals suggest reduced temperature variability in the eastern pole of the Indian Ocean Dipole during the medieval climate anomaly

**DOI:** 10.1038/s41598-021-94465-1

**Published:** 2021-07-22

**Authors:** Sri Yudawati Cahyarini, Miriam Pfeiffer, Lars Reuning, Volker Liebetrau, Wolf-Chr. Dullo, Hideko Takayanagi, Iwan Pramesti Anwar, Dwi Amanda Utami, Dieter Garbe-Schönberg, Marfasran Hendrizan, Anton Eisenhauer

**Affiliations:** 1 Paleoclimate & Paleoenvironment Research Group, Res. Cent. for Geotechnology-Indonesian Institute of Sciences (LIPI), Komplek LIPI Gd. 70-80 Jl Sangkuriang, Bandung, 40135 Indonesia; 2 RWTH Aachen University, Geology and Paleontology, 52056 Aachen, Germany; 3 Institute of Geosciences, Kiel University, 24118 Kiel, Germany; 4 GEOMAR Helmholtz Centre for Ocean Research Kiel, 24148 Kiel, Germany; 5 Institute of Geology and Paleontology, Graduate School of Science, Tohoku University, Sendai, Japan; 6 Department Oceanography, Institut Teknologi Bandung (ITB), Bandung, 40132 Indonesia

**Keywords:** Palaeoceanography, Palaeoclimate

## Abstract

We present two 40 year records of monthly coral Sr/Ca ratios from the eastern pole of the Indian Ocean Dipole. A modern coral covers the period from 1968 to 2007. A sub-fossil coral derives from the medieval climate anomaly (MCA) and spans 1100–1140 ad. The modern coral records SST variability in the eastern pole of the Indian Ocean Dipole. A strong correlation is also found between coral Sr/Ca and the IOD index. The correlation with ENSO is asymmetric: the coral shows a moderate correlation with El Niño and a weak correlation with La Niña. The modern coral shows large interannual variability. Extreme IOD events cause cooling > 3 °C (1994, 1997) or ~ 2 °C (2006). In total, the modern coral indicates 32 warm/cool events, with 16 cool and 16 warm events. The MCA coral shows 24 warm/cool events, with 14 cool and 10 warm events. Only one cool event could be comparable to the positive Indian Ocean Dipole in 2006. The seasonal cycle of the MCA coral is reduced (< 50% of to the modern) and the skewness of the Sr/Ca data is lower. This suggests a deeper thermocline in the eastern Indian Ocean associated with a La Niña-like mean state in the Indo-Pacific during the MCA.

## Introduction

**Figure 1:**
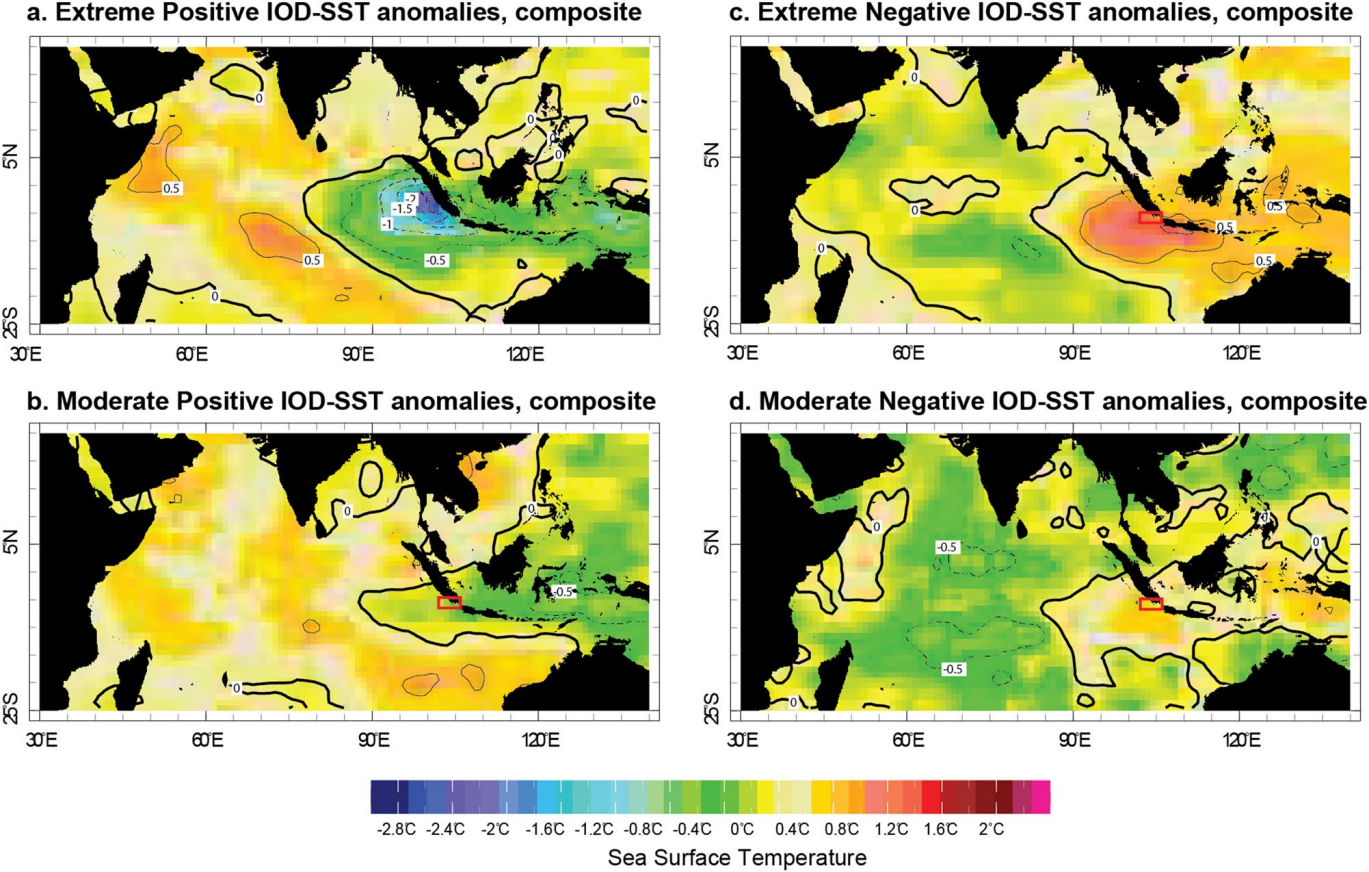
Composite maps of positive and negative IOD events. September–November SST anomalies during extreme (moderate) positive (**a**,**b**) and negative (**c**,**d**) IOD events. SST data is from the OISST v2 dataset. IOD events are classified according to the Australian Government Bureau of Meteorology (http://www.bom.gov.au/climate/iod/). Red rectangle includes Enggano Island (modern coral record) and Lampung Bay (MCA coral record). Map is generated using expert mode menu in https://iridl.ldeo.columbia.edu/SOURCES/.NOAA/.NCDC/.OISST/.version2/.AVHRR/.sst/.

The Indian Ocean Dipole (IOD) involves an aperiodic oscillation of sea surface temperatures (SST) in the equatorial Indian Ocean^1,2^. The IOD alternates between positive, neutral and negative phases. A positive IOD causes upwelling and cooling in the eastern equatorial Indian Ocean, off the coast of Java and Sumatra (Fig. 1), and droughts in adjacent land areas of Indonesia and Australia^3^. The western Indian Ocean warms and above-average precipitation occurs in equatorial East Africa^2,4^. The negative phase of the IOD causes opposite conditions, with warmer water and greater precipitation in the eastern Indian Ocean, and cooler and drier conditions in the west^1,2^. The IOD is asymmetric, a positive IOD tends to have stronger cold sea surface temperature anomalies over the eastern pole of the IOD (IODE; 90° E–110° E, 10° S–Eq.) than warm SST anomalies during its negative phase^5^ (Fig. 1). Positive IODs display strong inter‐event differences, with extreme events dominated by westward‐extended strong cold anomalies along the equator, and moderate events with weakened cooling confined to the region off Sumatra‐Java (Fig. 1a,b). In 2019, one of the strongest positive IOD events ever recorded caused an extreme drought over Indonesia and Australia, as well as flooding in equatorial East Africa followed by plagues of locusts.^6^

In the equatorial Pacific, the El Niño Southern Oscillation (ENSO), a similar, but stronger coupled ocean–atmosphere phenomenon operates. ENSO features a warm phase (El Niño), which is characterized by positive SST anomalies over the eastern Pacific and negative SST anomalies in the Western Pacific Warm Pool, including Indonesia and surrounding areas, and a cold phase (La Niña) with opposite conditions (Supplementary Fig. S1). ENSO dominates the interannual variability of global mean temperatures^7^, and also exerts a significant influence on the tropical Indian Ocean and Indonesia. However, many IOD events coincide with ENSO events^8–10^, and this may amplify their regional impact, for example in the South China Sea^11,12^.

Both ENSO and the IOD have severe socio-economic consequences, as they may cause serious droughts or floods over adjacent land areas. However, the response of the IOD and ENSO to future global warming is uncertain, as the instrumental record is too short to capture the full spectrum of their variability^13,14^. Coral oxygen isotope records from Sumatra, located in the eastern Indian ocean, show that extreme positive IOD events were rare before 1960^15,16^.

In Indonesia, ENSO and/or the IOD strongly influence SST and precipitation. However, the correlation differs across the region^17^. Moreover, the relationship between the Asian monsoon and ENSO/IOD is not well understood^18^. To better understand the impact of ENSO and IOD variability in Indonesia, we need to know how they varied in the past.

The Medieval Climate Anomaly (MCA) is a warm period lasting from approximately 900–1300 AD, with a core period from 950 to 1250 AD^19^, which was caused by natural changes^19–21^. It is still debated whether the MCA is a global phenomenon or more regional in extend^22^. A study from Ref.^19^ suggests that the MCA is a global phenomenon, and this is supported in recent studies^23,24^. Nevertheless, some regions have experienced cooling during this time^23,25^. Most paleoclimatic studies indicate warmer temperatures in Indonesia during the MCA, with a tendency towards La Niña-like conditions^19^, but the hydrological and oceanographic changes associated with this warming are not fully understood^26–28^, partly due to insufficient data coverage^26^.

Reconstructing the IOD and ENSO in the MCA is a challenge. Sub-fossil corals provide seasonal resolution and the most direct estimate of interannual phenomena such as ENSO and IOD during the past millennium^15,29,30^. However, while exceptionally long cores from living corals may span almost 500 years^31^, records from sub-fossil corals are typically much shorter and often only cover a few decades^30^. Also, dating uncertainties make it difficult to temporally align sub-fossil coral records from the eastern and western pole of the IOD. Hence, it is not possible to reconstruct the IOD index, which captures the SST difference between the eastern and western tropical Indian Ocean, from sub-fossil corals. It has been shown, however, that IOD variability can be reconstructed from sub-fossil corals that derive from the eastern pole of the IOD (IODE, 90° E–110° E, 10° S–Eq)^28,30^. These single-site coral records capture up to 50% of IOD variance. An analysis of climate model data also supports the assumption that the sea surface temperature–IOD relationship in the IODE region is stationary over time. This is necessary for paleoclimate reconstructions^32^. Thus, corals from the eastern pole of the IOD provide a record of IOD variability during the past millennium and its interaction with ENSO^15^. At present, this record is still incomplete and based on coral δ^18^O measurements, which record both temperature and δ^18^Osw, the latter influenced by the precipitation/evaporation balance and/or oceanic advection^15^. Coral Sr/Ca, in contrast, is solely a temperature proxy. The Sr/Ca ratios of coral aragonite skeletons provide monthly resolved records of past temperature variations^33–39^ that are not influenced by changes in the hydrological cycle.

A well-known feature of the IOD is its skewness, whereby positive IOD events tend to grow much larger than negative IOD events (so that the IOD is positively skewed). Observations suggest that the positive IOD skewness primarily reflects the negative SST skewness in IODE SST, as the western pole of the IOD exhibits only a weak positive SST skewness^40,41^. The negative skewness of IODE is caused by a positive Bjerkness feedback involving the SST response to the depth of the thermocline in the eastern Indian Ocean: cold IODE SST anomalies lead to a zonal SST gradient that drives an easterly wind anomaly in the equatorial Indian Ocean, which further shoals the thermocline in the eastern Indian Ocean, reinforcing the cold SST anomalies there^5,14,40^.

**Figure 2:**
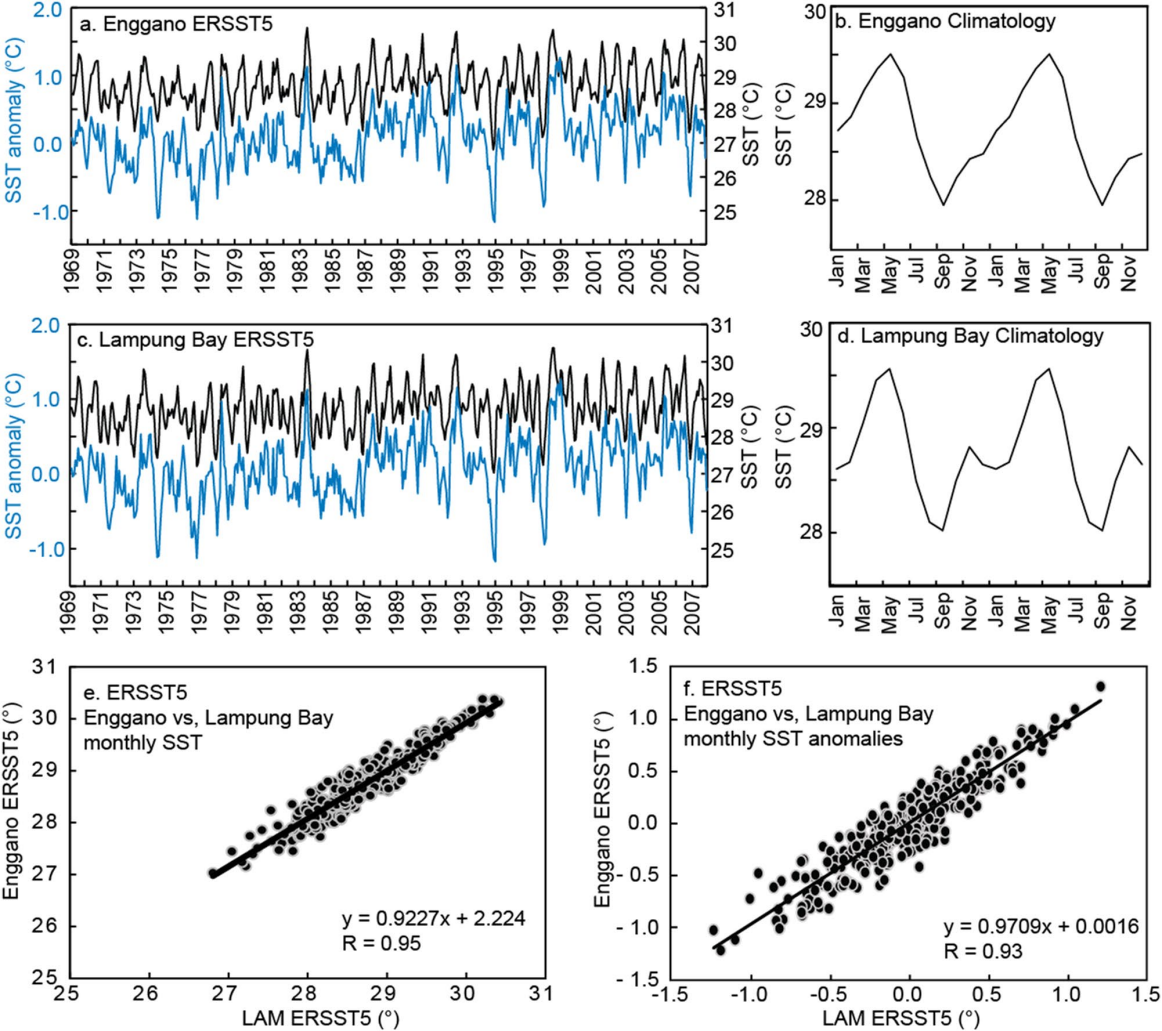
Sea surface temperature variability at Enggano Island and Lampung Bay. (**a–d**) (left) Monthly SST (black lines), its anomaly (blue lines) and (right) its climatology. (**e**) Linear regression of monthly SST from Enggano Island versus SST from Lampung Bay. (**f**) Same as (**e**) but for monthly anomalies. SST data is from ERSSTv5^49^ from the grid boxes including the coral sites, and displayed over the period of Nov 1968–Sep 2007: (**a**,**b**) Enggano Island (102.125 E 5.375 S) and (**c**,**d**) Lampung bay, Sunda strait (105.578 E 5.749 S).

**Figure 3:**
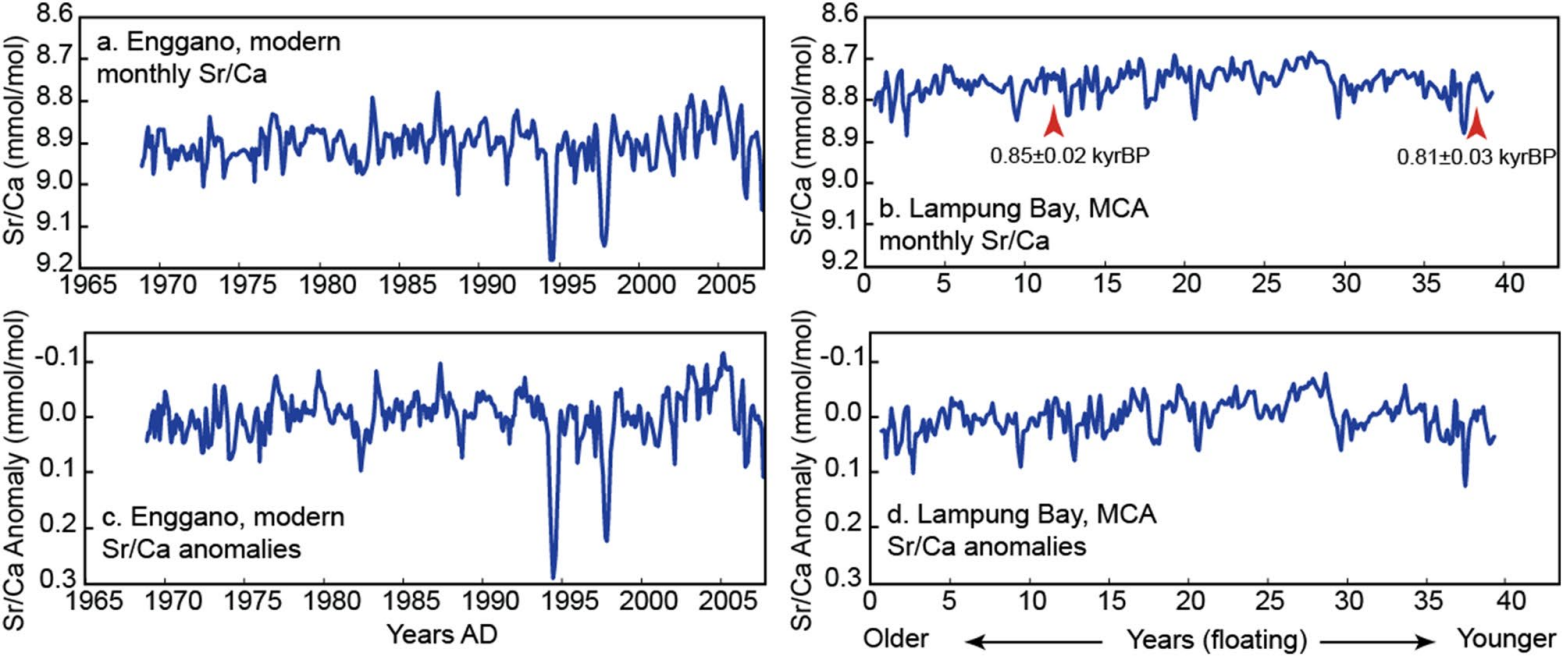
Modern and MCA coral Sr/Ca time series. (**a**) Monthly coral Sr/Ca record of the modern coral from Enggano Island (KN2). (**b**) Monthly coral Sr/Ca record of the MCA coral from Lampung Bay. Red arrows mark the U/Th dates. (**c**) Monthly anomalies of the modern coral from Enggano Island. (**d**) Same as (**c**) but for the MCA coral from Lampung Bay.

In this study, we present two 40-year reconstructions of seasonal SST variability based on monthly coral Sr/Ca data from the IODE region. The corals were collected at Enggano Island and Lampung Bay. Both sites are located in south western Indonesia, off the coast of Sumatra, and face the south eastern Indian Ocean (Supplementary Fig. S2a). At both sites, SSTs covary (Fig. 2) and SSTs are strongly influenced by the IOD with cooling during moderate and strong positive IOD (pIOD) events, and warming during negative IOD (nIOD) events (Fig. 1). The Enggano Island Sr/Ca record derives from a modern core drilled in 2007 (KN2: 1968–2007 AD; 102.125 E, 5.375 S) (Fig. 3). The Lampung Bay Sr/Ca record derives from a sub-fossil coral (LAM: 1100–1140AD ± 25 years; 105.578 E, 5.749 S), which has been dated via U/Th (see “Methods” section) and derives from the MCA (Fig. 3). This study aims to compare present and MCA temperature variability in the IODE region inferred from monthly coral Sr/Ca ratios, assesses the frequency and magnitude of interannual SST anomalies attributable to the IOD and/or ENSO, and the asymmetry of SST in the IODE region.

## Results

### Climate and oceanic setting of the study area

Enggano Island is located in the Indian Ocean, west of southern Sumatra. Lampung Bay is located in Sunda Strait, between Sumatra and Java (see Supplementary Fig. S2). During the Southeast (SE) monsoon, zonal winds trigger coastal upwelling off southern Java, which propagates northwards along the Java-Sumatra coast and causes a drop in SST (e.g. Ref.^42^). Low-salinity, and low-density waters from the Java Sea are exported to the Indian Ocean through Sunda Strait, enhancing the Indonesian Throughflow (ITF) transport from the Pacific into the Indian Ocean^43^. Positive IOD events enhance the southward Sunda Strait flow due to strong anomalous easterly winds along the coast of Java^1,28,43^. Furthermore, the ITF is weaker during El Niño and stronger during the La Niña events, with Niño 3.4 leading the ITF by 7 months^44^. During the North West (NW) monsoon, water from the Indian Ocean is transported through Sunda Strait, from where it then moves eastward and cools the Java Sea^43,45–48^. Thus, ocean advection may influence δ^18^O_sw_ and hence coral δ^18^O. We therefore decided to use the coral Sr/Ca thermometer to reconstruct SST variability.

At Enggano Island and Lampung Bay, seasonal variations of SST are strongly influenced by the Asian-Australian monsoon^43,45,47,48^. At both sites, moderate to strong positive (negative) IOD events cause cold (warm) SST anomalies (Fig. 1).

### Instrumental SST data: seasonality and signature of IOD and ENSO events

The Extended Reconstructed Sea Surface Temperature (ERSSTv5) (Fig. 2)^49^ from the grids including Lampung Bay and Enggano Island is used to describe the climatology of the study area and to compare the SST variability at both sites. Climatological SST data is calculated by taking the monthly mean of SST for the period from Nov 1968 to Sep 2007. At Enggano, the mean seasonal cycle of SST is 1.6 °C, with maximum (minimum) SSTs in May (September) of 29.5 °C (28.0 °C) (Fig. 2b). Climatological SST shows that SST at Lampung Bay has a mean seasonal cycle of 1.5 °C, with maximum SST in May (29.6 °C) and minimum SST in September (28.0 °C) (Fig. 2d). Time series of monthly means, monthly anomalies and annual means of historical SSTs from both sites are highly correlated (R_monthly_ = 0.93–0.95 N = 311, Fig. 2e,f, R_annual_ = 0.95–0.97 N = 40, Supplementary Fig. S3d), and both sites show the same cooling during positive IOD events (Fig. 2, Supplementary Fig. S3). This demonstrates that SST variability at both sites covaries on seasonal, interannual and longer time scales.

To investigate the signature of the IOD and ENSO in SST at our coral sites, instrumental SST is correlated with various climatic indices (see “Methods” section). The Niño 3.4 index is an SST anomaly index from the equatorial Pacific averaged over 5° N–5° S, 120–170° W that captures interannual ENSO variability. The Dipole Mode Index (DMI) or IOD index is the difference between SST anomalies in the western and eastern tropical Indian Ocean (IOD west: 50° E to 70° E; 10° S to 10° N; IOD east: 90° E to 110° E; 10° S to 0° S). IODE index is the SST anomaly index averaged over the eastern pole of the IOD (note that positive IOD events register as positive anomalies in the DMI index and negative anomalies in IODE). Niño 3.4, DMI and IODE index are taken from ERRSTv5 data.

Results show that the correlation of instrumental SST from Enggano Island and Lampung Bay with the Niño 3.4 and the DMI depends on the season. The Niño 3.4-SST correlation changes from negative in boreal summer and fall (SE monsoon season) to positive in boreal winter and spring (NE monsoon season). The correlation with the DMI is stronger, but it is only significant in boreal summer and fall, when IOD events develop and peak. The correlation with IODE index is strong and stable throughout all seasons (Supplementary Fig. S4), which is to be expected as IODE index is a large-scale SST average in the eastern Indian Ocean that includes Lampung Bay and Enggano Island. This shows that the SST-ENSO (DMI) relationship varies depending in the phase of the SE Asian monsoon, while the correlation with IODE index is stable throughout the year.

### Modern coral Sr/Ca data: calibration and signature of IOD and ENSO events

**Figure 4:**
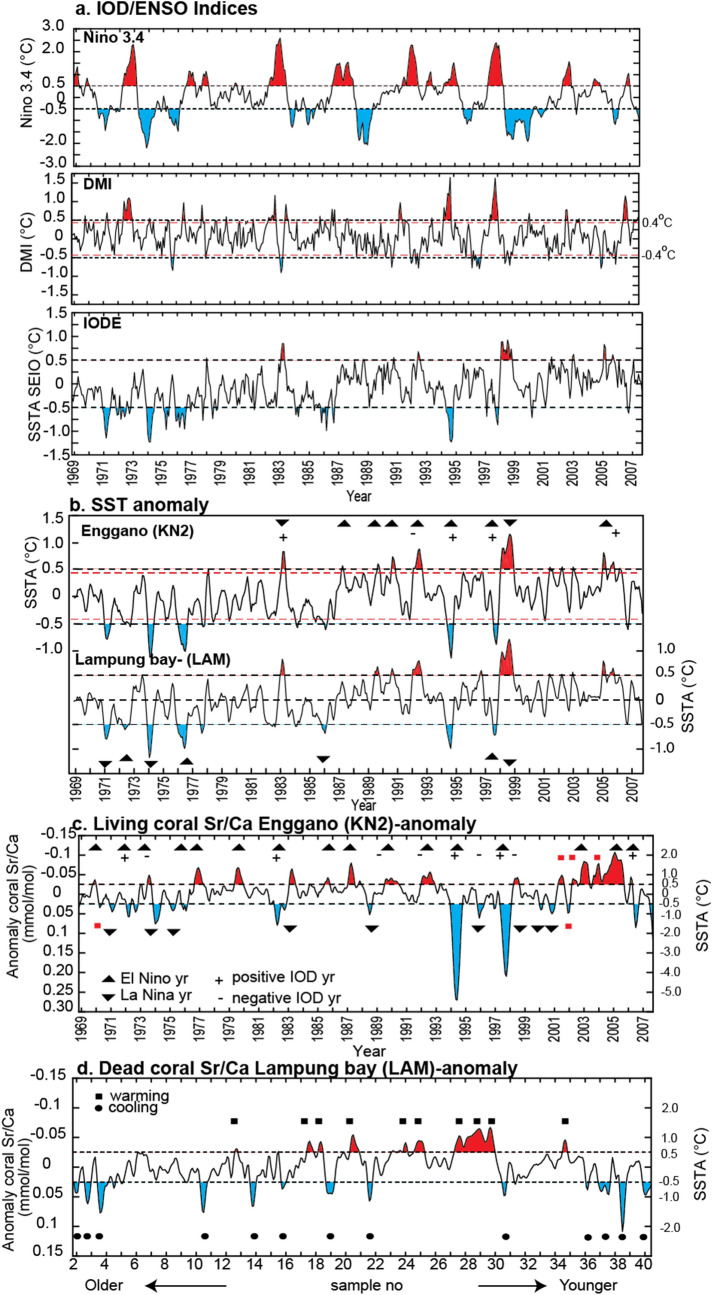
ENSO and IOD variability, modern and MCA. (**a**) Nino 3.4, DMI and IODE indices based on ERSST v 5 accessed via the KNMI climate explorer (https://climexp.knmi.nl/) (**b**) SST anomaly records (3 months running means, ERSST v 5) from the grids including Enggano Island and Lampung bay. (**c**,**d**) Coral Sr/Ca anomalies (3 months running means) of (**c**) the modern coral record from Enggano Island (KN2) and (**d**) the MCA coral from Lampung bay-Sunda strait (LAM). In (**c**) IOD events are indicated (based on the list provided by the Australian Bureau of Meteorology: http://www.bom.gov.au/climate/iod/). Plus sign: positive IOD events; minus sign: negative events. El Nino (triangle pointing up) and La Nina events (triangle pointing down) are taken from the NOAA climate prediction centre-Ocean Nino Index (ONI; https://origin.cpc.ncep.noaa.gov/products/analysis_monitoring/ensostuff/ONI_v5.php). In (**c**) and (**d**) a threshold of ± 0.025 mmol/mol indicates Sr/Ca anomalies that exceed 0.4–0.5 °C. Note that most of these events in the modern coral record [shown in (**c**)] can be attributed to either the IOD or ENSO (red squares mark events that cannot be attributed to the IOD or ENSO). In the MCA coral record shown in (**d**), Sr/Ca anomalies below (above) the threshold of ± 0.025 mmol/mol are indicated by black dots (black squares) and interpreted as IOD/ENSO-type events. See text for discussion.

The modern coral Sr/Ca record is shown in Fig. 4 as monthly data and as monthly anomalies. The modern coral Sr/Ca record from Enggano Island (KN2) is calibrated with ERSSTv5 data from the grid including Enggano Island. Results show a high linear correlation (monthly means and monthly anomalies: R = 0.50–0.68, N = 311, *p* ≤ 0.0001, 95% confidential level; annual means: R = 0.50–0.75 N = 27–40,* p* ≤ 0.0001, 95% confidential level) (see Supplementary Fig. S5). Slope values are consistent with published Sr/Ca–SST relationships that range from − 0.04 to − 0.08 mmol/mol per 1 °C^34,36,37,50^. Calibrating the Enggano Sr/Ca record with ERSSTv5 from the neighboring grid, which includes Lampung Bay, shows similar correlation coefficients and regression parameters (Supplementary Fig. S5c,d).

The monthly mean modern coral Sr/Ca record from Enggano Island is correlated with the Niño3.4 and DMI, as well as with IODE index (Supplementary Figs. S4, S6). The coral data shows the same seasonal correlation pattern as the instrumental data (with reversed sign due to the negative Sr/Ca–SST dependence). The Niño 3.4 correlation changes in sign from negative in July–October to positive in December–February (Supplementary Figs. S4, S7), while the correlation with the IOD index is strongest in July–October (Supplementary Fig. S4). The correlation with IODE index is stable throughout the year, although slightly lower compared to instrumental data, reflecting the noisiness of the proxy data (Supplementary Figs. S4, S7e,f). A spatial correlation analysis of the September–November mean Enggano Sr/Ca record shows the typical IOD pattern, with cooling in the eastern pole of the IOD (including Lampung Bay) and warming in the west (Supplementary Fig. S7).

To further explore these relationships, we select positive/negative IOD events (DMI and IODE index) and warm/cold ENSO events and correlate them with the corresponding coral Sr/Ca anomalies (Supplementary Fig. S6). Positive and negative IOD events are taken from the list of the Australian Government Bureau of Meteorology (http://www.bom.gov.au/climate/iod/), and ENSO warm/cold events are taken from the US Climate Prediction Centre (https://origin.cpc.ncep.noaa.gov). The results show that the correlation between the modern coral Sr/Ca anomaly record and the DMI (N = 49, R = 0.53–0.56, *p* ≤ 0.0001) is strong, is the correlation with the IODE index (N = 49, R = 0.49–0.63, *p* ≤ 0.0001) (Supplementary Fig. S6a–d). The coral records both negative and positive IOD events. The correlation with El Niño events is also strong (n = 49, R = 0.42, *p* ≤ 0.0001), while the coral Sr/Ca record does not record La Niña events (n = 49, R = 0.10, *p* = 0.42, not significant) (Supplementary Fig. S6e,f). The strongest correlation is found between modern coral Sr/Ca data and IODE index during positive IOD events (n = 49, R = 0.63, *p* ≤ 0.0001) (Supplementary Fig. S6b). This is expected as the coral Sr/Ca record from Enggano Island derives from the IODE region and faithfully records SST variability in that region.

Having established that the modern coral Sr/Ca record from Enggano Island faithfully records interannual SST variability associated with ENSO and the IOD, we independently identify IOD/ENSO events in the modern coral Sr/Ca record from Enggano based on the magnitude of the Sr/Ca anomaly in that core (Fig. 4). We select a threshold of ± 0.025 mmol/mol (± 0.4–0.5 °C in terms of SST anomalies assuming a Sr/Ca–temperature relationship of − 0.05 to − 0.06 mmol/mol per 1 °C, as estimated via the modern coral Sr/Ca–SST calibrations). IOD/ENSO years are regarded as ‘not recorded’, if the Sr/Ca anomaly does not exceed the threshold of ± 0.025 mmol/mol. This is somewhat arbitrary, however there is currently no consensus on the classification of IOD events. The Australian Government Bureau of Meteorology lists positive/negative IOD events when the DMI index exceeds ± 0.4 °C, but various IOD reconstructions report different numbers of IOD events^51^ and there are currently no reconstructions of IOD variability that are based solely on SST anomalies in the IODE region. Note that we did not filter the Sr/Ca record, so that the full spectrum of SST variability (including decadal variability) is retained.

Table 1 lists the cool/warm SST anomalies recorded in Enggano coral Sr/Ca and the corresponding phase of the IOD. We also indicate the state of ENSO corresponding to the IOD events. Positive IOD years which are recorded as a cool anomaly in the modern coral Sr/Ca anomaly from Enggano are: 1972, 1982, 1983, 1994, 1997, 2006. The magnitude of the cooling differs markedly. The cooling during the extreme IOD events of 1994 and 1997 exceeds − 3 °C, while the IOD of 2006 led to a maximum cooling of approximately − 2 °C (Supplementary Fig. S3). The cooling reported during these extreme positive IOD events is consistent with satellite SST data that extend back until 1982 (see Supplementary Figs. S3 and S5). Note that the ERSSTv5 data shown in Fig. 2 and Supplementary Fig. S3 tends to underestimate the cooling during extreme positive IOD events as these are amplified by non-linear oceanic feedbacks; see Supplementary Fig. S4 in Ref.^52^. During moderate positive IOD events, the magnitude of cooling indicated by coral Sr/Ca is between − 1 and − 2 °C, consistent with satellite data (Figs. 3c,d).

**Table 1:** IOD response seen in the Enggano coral record.

Recorded-IOD events	Type		SST response at Enggano as recorded in coral	Recorded ENSO events
**1972**	+	7	Cooling	Weak La Nina
**1974**	−	7	Warming	Weak La Nina
1981	−	X	Warming, below the threshold	−
*1982*	+	7	Cooling	Very strong El Nino
*1983*	+	7	Cooling	Very strong El Nino
1989	−	X	Warming, below the threshold	Strong La Nina
*1992*	−	7	Warming	Strong El Nino
*1994*	+	7	Cooling	Moderate El Nino
**1996**	−	X	Cooling	Moderate La Nina
*1997*	+	7	Cooling	Very strong El Nino
**1998**	−	7	Warming	Strong La Nina
*2006*	+	7	cooling	Weak El Nino

7: IOD/ENSO events and response seen in the Enggano coral record.

Bold: the La Nina years.

Italics: the El Nino years.

Black (X): not recorded/no event.

During negative IOD or La Niña years, anomalous warming usually occurs off south western Sumatra^53,54^. Negative IOD years recorded by a negative (= warm) anomaly in coral Sr/Ca are 1974, 1992 and 1998 (Fig. 4, Table 1). Warming in 1981 and 1989 remains below the threshold of − 0.025 mmol/mol (Fig. 4, Table 1) and 1996 does not show the expected warming typical for negative IODs. Contrary to expectations, the coral Sr/Ca record shows anomalous cooling during the negative IOD event of 1996. However, the cooling anomaly during 1996 shown in the Enggano coral is also shown in SST data (see Supplementary Fig. S8 for more details). The cooling anomaly starts in November 1995 to March 1996, i.e. in the NW monsoon seasons, which precedes the IOD season which usually develops in boreal summer and decays in boreal winter^55^. Abrupt warming in April 1996 likely reflects the influence of the ensuing negative IOD. Warming persists until November 1996, but remains below the threshold for negative IOD events (see Supplementary Fig. S8 for more detail).

Table 2 compares Enggano coral Sr/Ca anomalies with ENSO events. There are only few ENSO events which are not recorded in the coral Sr/Ca record, i.e. the El Niño event of 1991/92, and the La Niña events of 1984/85 and 1999/2000 (Table 2). During these events the Sr/Ca anomaly does not exceed the threshold of ± 0.025 mmol/mol (which corresponds to a SST anomaly of ± 0.5 °C). In total, there are 32 warm and cool events recorded in the modern coral Sr/Ca record from Enggano Island that can be attributed to the IOD and/or ENSO. Note that mean SSTs appear to be a little colder prior to 1975 (Fig. 2) and may reflect decadal variability associated with the well-known regime shift in the tropical Pacific^56^. However, given the pronounced interannual variability in the coral record from Enggano Island, the statistical significance of this shift is difficult to evaluate. Some SST anomalies recorded in the modern coral Sr/Ca record are not explained by IOD and/or ENSO (e.g. the warm years in 2004/2005 and 2002/2003, see Fig. 4). However, most interannual Sr/Ca anomalies exceeding the threshold of ± 0.025 mmol/mol are related to IOD and/or ENSO events.

**Table 2:** ENSO years and their SST signature in the Enggano Sr/Ca record.

Coral indicates cooling			Coral indicate warming		
ENSO years recorded in coral	Degree of events	Remark	ENSO years recorded in coral	Degree of events	Remark
**1970–71**	Moderate La Nina	٧	*1969–70*	Weak El Nino	٧
**1971–72**	Weak La Nina	٧	**1973–74**	Strong La Nina	٧
*1972–73*	Strong El Nino	٧	*1977–78*	Weak El Nino	٧
**1974–75**	Weak La Nina	٧	*1980–81*	Weak El Nino	Known ENSO years is 1979–80 (weak El Nino), but in coral the SST anomaly is found in 1980–81
**1975–76**	Strong La Nina	٧	**1983–84**	Weak La Nina	
*1976–77*	Weak El Nino	٧	*1985–86*	Moderate El Nino	Known ENSO years is 1986–87 (moderate El Nino), but in coral the SST anomaly is 1985–86
*1982–83*	Very strong El Nino	٧	*1987–88*	Strong El Nino	٧
**1988–89**	Strong La Nina	٧	1990–91	Normal	There is warming SST anomaly indicate in coral but it is known normal years (no ENSO)
*1994–95*	Moderate El Nino	٧	1992–93	Normal	
**1996–97**	Moderate La Nina	Known ENSO is 1995–96, But in coral the SST anomaly is found in 1996–97	**1998–99**	Strong La Nina	٧
*1997–98*	Very strong El Nino	٧	2001–02		
**2000–01**	Weak La Nina	٧	2003–04		
*2002–03*	Moderate El Nino	٧	*2004–05*	Weak El Nino	٧
*2006–07*	Weak El Nino	٧	**2005–06**	Weak	٧

*1991–92*: Strong El Nino.

**1984–85**: Weak La Nina.

**1999–00**: Strong La Nina.

### Modern and MCA coral Sr/Ca: time series and descriptive statistics

Figure 3 shows the monthly resolved modern and MCA coral Sr/Ca records from Enggano Island and Lampung Bay, respectively. Both records are plotted on the same scale, as absolute Sr/Ca values and as monthly anomalies with their mean seasonal cycles removed. Compared to the modern data, the MCA record shows reduced variability and more negative mean Sr/Ca values, which would indicate warmer mean temperatures (if the offset in mean Sr/Ca is temperature related; see discussion) and reduced SST variability.

Table 3 compares basic statistics of the modern (KN2) and MCA (LAM) coral Sr/Ca data. The mean Sr/Ca ratio of KN2 is 8.903 ± 0.003 mmol/mol (N = 420, raw data), while the mean Sr/Ca ratio of LAM is 8.758 ± 0.002 mmol/mol (N = 239, raw data). The min/max values are 8.766/9.180 mmol/mol (KN2, N = 420) and 8.682/8.891 mmol/mol (LAM, N = 239). The standard deviation of the raw Sr/Ca data is 0.054 mmol/mol (KN2) and 0.036 mmol/mol (LAM), i.e. the spread around the mean reduces to ~ 67% of modern values in the MCA. The sampling resolution of KN2 is higher but this does not influence the standard deviation (re-sampling KN2 to N = 210 by averaging two adjacent Sr/Ca values does not significantly reduce the standard deviation).

**Table 3:** Descriptive statistic of raw data of coral Sr/Ca from MCA (LAM) and Enggano (KN2) samples.

KN2 monthly		LAM monthly	
Mean	8.903	Mean	8.758
Standard error	0.003	Standard error	0.002
Median	8.900	Median	8.755
Standard deviation	0.054	Standard deviation	0.036
Sample variance	0.003	Sample variance	0.001
Kurtosis	5.047	Kurtosis	1.150
Skewness	1.301	Skewness	0.790
Range	0.415	Range	0.209
Minimum	8.766	Minimum	8.682
Maximum	9.180	Maximum	8.891
Sum	3739	Sum	2093
Count	420	Count	239
Confidence level (95.0%)	0.005	Confidence level (95.0%)	0.005

KN2 annual		LAM annual	
Mean	8.907	Mean	8.756
Standard error	0.005	Standard error	0.004
Median	8.906	Median	8.763
Standard deviation	0.034	Standard deviation	0.024
Sample variance	0.001	Sample variance	0.001
Kurtosis	3.183	Kurtosis	0.255
Skewness	0.873	Skewness	0.465
Range	0.193	Range	0.101
Minimum	8.835	Minimum	8.700
Maximum	9.028	Maximum	8.801
Sum	356	Sum	350
Count	40	Count	40
Confidence level (95.0%)	0.011	Confidence level (95.0%)	0.008

At annual mean resolution, the mean Sr/Ca ratio of KN2 is 8.907 ± 0.005 mmol/mol (N = 40). The mean Sr/Ca ratio of LAM is 8.756 ± 0.004 mmol/mol (N = 40). Min/max values of the annual means are 8.835/9.028 mmol/mol (KN2) and 8.700/8.801 mmol/mol (LAM) (Table 3).

We analysed the skewness of the non-interpolated monthly coral Sr/Ca data. The results show that the modern coral Sr/Ca record (skewness 1.449 ± 0.671 mmol/mol, N = 365, significant at the 95% confidence interval) is more skewed towards positive Sr/Ca values (indicating cold temperatures) than the MCA coral (skewness 0.787 ± 0.338, N = 239). In climate studies, the skewness has been used as a measure of temperature variability^57,58^, to search for abrupt changes in temperature^59^, or to obtain information on the amplitude of climate events^60^. The skewness provides a measure of the asymmetry of the coral Sr/Ca data, which reflects the asymmetry of SST anomalies in the IODE region. The LAM Sr/Ca record suggests that IODE SST variability was less skewed towards cold SST anomalies in the MCA. This would suggest fewer extreme positive IOD events.

### Modern and MCA coral Sr/Ca data: seasonal and interannual variability

**Figure 5:**
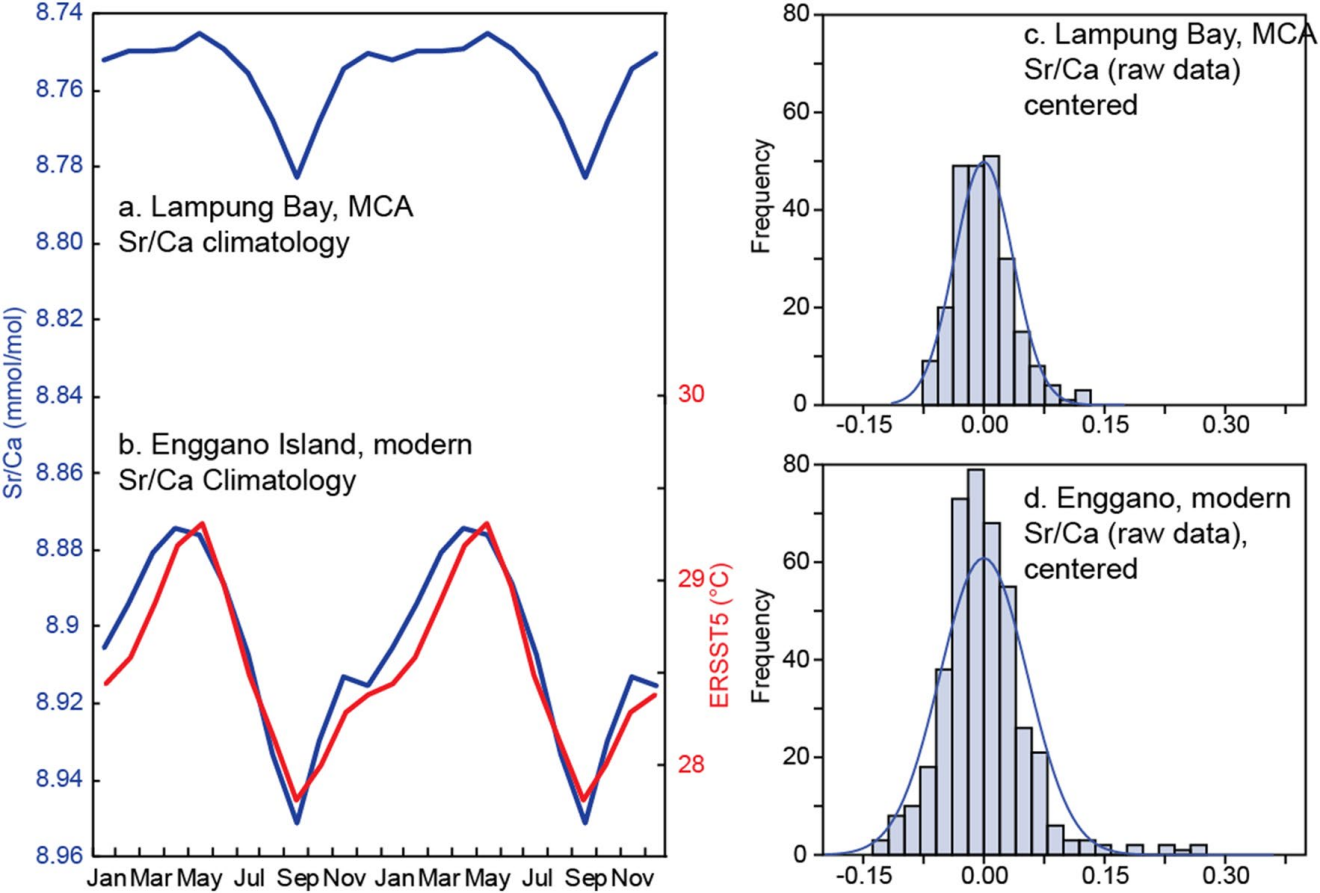
Seasonality and skewness of modern and MCA coral Sr/Ca data. (**a**) Climatological Sr/Ca data calculated from the monthly record from Lampung Bay (LAM). (**b**) Same as (**a**), but calculated from the modern record from Enggano Island (blue line). Red line shows ERSST5 climatology for comparison. (**c**) Histogram of Lampung bay Sr/Ca (raw data) and (**d**) histogram of Enggano Island Sr/Ca (raw data, centred by removing its mean). Note the skewness of the Enggano data that reflects the pronounced cooling during extreme positive IOD events (see Table 3 for basic statistics).

The mean seasonal cycle of coral Sr/Ca in the modern coral (KN2, Enggano Island) is 0.076 mmol/mol (Fig. 5). The sub-fossil coral from Lampung Bay has a mean seasonal cycle of 0.038 mmol/mol (Fig. 5), suggesting a reduction in temperature seasonality up to 50% during the MCA. The reduction in seasonality coincides with the reduced skewness of the Sr/Ca data (Fig. 5, Table 3), which is to be expected as extreme positive IOD events tend to inflate the seasonal cycle and contribute to the skewness of IODE SST (see “Discussion”).

The MCA coral from Lampung Bay shows a number of coral Sr/Ca anomalies exceeding the threshold of ± 0.025 mmol/mol (corresponding to 0.4–0.5 °C given our Sr/Ca–SST relationships of − 0.05 to − 0.06 mmol/mol/°C). These anomalies are comparable to the IOD/ENSO signature in the modern coral Sr/Ca record from Enggano Island. We therefore attribute these anomalies to IOD and/or ENSO events that occurred during the medieval climate anomaly (Fig. 4). There are 24 warm/cool events inferred from the Sr/Ca anomaly record of the MCA coral from Lampung Bay, fewer than in the modern coral Sr/Ca record from Enggano Island. There are more cool events (14) than warm events (10) (Fig. 4). However, the magnitude of the cool events remains between − 1 and − 2 °C, with the exception of one anomaly in year 38 that reaches − 2 °C, which would approximately correspond to the extreme positive IOD event of 2006 (Note, however, that this event may be inflated by decadal variability in the LAM Sr/Ca record). Extreme positive events on par with the events of 1994 and 1997 (or 2019, which also caused cooling exceeding-3 °C in OI SST data) are not recorded in the sub-fossil coral from Lampung Bay (Fig. 4). Warm Sr/Ca anomalies are between 1–2 °C, slightly less than in the modern record from Enggano Island.

## Discussion

Previous coral reconstructions of the IOD are based on coral δ^18^O, which records both temperature and δ^18^O seawater. Even a small influence of δ^18^O seawater on coral δ^18^O will bias δ^18^O–SST reconstructions^61^. Indonesia has an intense hydrological cycle driven by the monsoon and mixing of water masses due to oceanic advection, and these processes all influence δ^18^O seawater^15,62–64^. Hence, coral δ^18^O reconstructions from the eastern pole of the IOD can be used to reconstruct IOD variability, but not SST variability (see Ref.^15^ for a summary of sub-fossil IOD reconstructions based on coral δ^18^O). While rainfall often co-varies with SST and enhances the coral δ^18^O–SST correlation, oceanic processes such as advection and upwelling also influence δ^18^O seawater and salinity and do not necessarily co-vary with SST^65^. At sites like Lampung Bay, which is located in Sunda Strait, where advection of water masses from the Java Sea and the Indian Ocean is important, coral δ^18^O should be interpreted with caution. In contrast, coral Sr/Ca provides a pure temperature proxy, and SSTs inferred from coral Sr/Ca reflect ocean–atmosphere interactions. In Indonesia, coral Sr/Ca often has a better SST correlation compared to coral δ^18^O alone^35,63^.

Here, we provide temperature reconstructions based on coral Sr/Ca for two 40-year windows from the eastern pole of the IOD. A modern core is used to investigate how coral Sr/Ca responds to the IOD and ENSO, and a sub-fossil coral (dated to 1100–1140 ad by U/Th) is used to infer changes in SST variability during the MCA. Spatial correlation and linear regression analysis confirm that the Enggano and Lampung Bay coral Sr/Ca records both show SST in the IODE region and can be used to compare present-day and MCA SST variability (see Supplementary Figs. S5, S7).

The modern coral Sr/Ca record reflects SST variability in the eastern pole of the IOD as expected based on the known Sr/Ca-temperature dependence. This includes the magnitude of the seasonal cycle of SST (Fig. 5) and the cooling (warming) during positive (negative) IOD events (Fig. 4). The asymmetry of the IOD, with pronounced cooling during positive IOD events compared to moderate warming during negative events, is reflected in the skewness of the modern Sr/Ca data. The ENSO correlation shows a shift from positive (= cooling during El Niño) in July–September to negative (= warming during El Niño) in the following boreal spring (January–March), which is typical for this region of Indonesia. The coral records the majority of, but not all, positive and negative IOD events, as well as El Niño events (La Niña events are few and mostly weak to moderate in the time interval covered by the modern coral Sr/Ca record; hence the correlation is weak). However, the interaction between the IOD and ENSO may also result in smaller than expected SST anomalies in some years (e.g. during the negative IOD of 1996). Decadal variability may also influence the magnitude of the interannual SST anomalies inferred from the data, although the modern record is dominated by interannual variability, as confirmed by power spectrum analysis (Supplementary Fig. S9).

Compared to the modern coral, the MCA coral shows a marked reduction in seasonality by almost 50%, from 1.5 to 0.7 °C (Fig. 5). Present-day temperature seasonality off Sumatra varies from ~ 1.5 °C at 5° S to ~ 1 °C at the Equator^49,66^, and largely depends on the strength of the alongshore winds during the SE monsoon, which drive coastal upwelling and cooling in July–September. However, the seasonality is influenced by interannual variability associated with the IOD that tends to inflate the seasonal cycle (omitting the IOD years listed by the Australian Government Bureau of Meteorology from the Enggano coral Sr/Ca record reduces its seasonality to ~ 1 °C). The standard deviation of the raw Sr/Ca data from the MCA coral also reduces when compared to the modern coral, by approximately 65–70%. The skewness reduces as fewer positive Sr/Ca extremes are present which would indicate the pronounced cooling typical for extreme positive IOD events. Also, the MCA coral Sr/Ca record does not show any clear extreme positive IOD events. One event is on par with the 2006 event, but this event may be inflated by decadal variability. Interannual variability exceeding the threshold of ± 0.025 mmol/mol, as expected during moderate IOD events and/or ENSO events, is present, but there are fewer events than in the modern coral. Power spectrum analysis does not show significant interannual variability in the MCA coral record (Supplementary Fig S9). Taken together, this suggests a reduction in interannual variability driven by the IOD and/or ENSO, and fewer extreme cold anomalies which contribute to a reduction in the skewness if IODE SSTs and a reduction in seasonality during the MCA.

**Figure 6:**
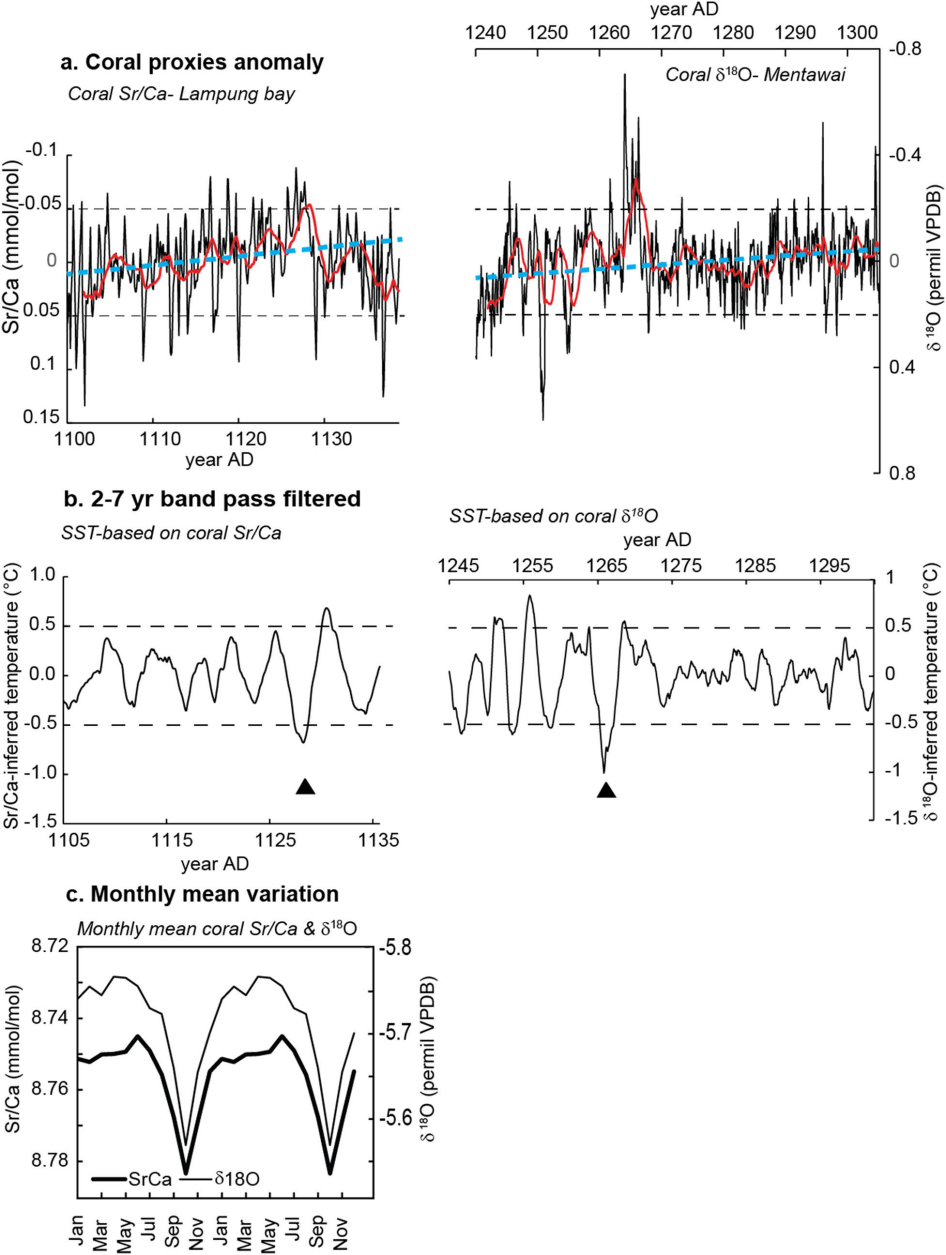
MCA coral proxy time series from the eastern pole of the IOD. (**a**) Coral Sr/Ca anomalies from Lampung Bay (LAM, this study) and coral δ^18^O anomalies from Mentawai^28^. Coral δ^18^O is an average of 3 colonies, which have been spliced together to extend the time series to 65 years. Anomaly data is calculated by removing the seasonal mean. Both proxies show a long-term warming trend during the MCA (blue dashed line). The slope of the linear trend is 0.0005 ± 0.0012 ‰ (for coral δ^18^O) and 0.0008 ± 0.0005 mmol/mol/°C (for coral Sr/Ca). Red line is 2 years moving average. Dashed black lines indicate threshold beyond which proxy anomalies exceed ± 1 °C (**b**) 2–7 years band pass filtered coral proxy data. The filtered data is converted to SST assuming a coral Sr/Ca–SST relationship of 0.05 mmol/°C and coral δ^18^O–SST relationship of 0.19 permil/°C. Arrow marks extreme IOD event (**c**) Monthly climatology of coral Sr/Ca (bold dark line) and δ^18^O (thin dark line).

We compare our results with a 65-year coral δ^18^O record from the southern Mentawai Islands dated to 1239–1305 ad (Fig. 6)^28^. We filtered the proxy records with a 2–7 years bandpass filter. For comparison, the filtered data is converted to SST units using published proxy–SST relationships, i.e. − 0.05 mmol/mol/°C for Sr/Ca and − 0.19 permil/°C for coral δ^18^O. The Mentawai record shows one extreme IOD event (> 1 °C) (Fig. 6b)^26^ while the largest anomaly recorded in LAM is ~ 0.7 °C. Both proxy records suggest a reduction in IOD variability compared to modern climate^28^. Coral Sr/Ca and δ^18^O climatologies suggest a similar seasonal cycle during the MCA, with a broad warm season (Fig. 6c). The seasonal cycle of the Mentawai δ^18^O record is slightly larger than the seasonal cycle of LAM Sr/Ca, which either reflects increased seasonality between 1239 and 1305 ad or an enhanced hydrological cycle with a warmer and wetter NW monsoon season. (Note that Mentawai is located further north compared to Enggano Island and Lampung Bay, and present-day seasonality is lower).

Our new coral Sr/Ca record from Lampung bay provides the first monthly resolved proxy record from the core period of the MCA, and extends the existing MCA record of IOD variability by 65%. However, taken together, the Mentawai and Lampung Bay records only cover ~ 26% of the period from 900 to 1300 AD. Therefore, a longer coral record from the MCA would be desirable to capture the full spectrum of variability in the eastern Indian Ocean, especially since the Lampung bay and Mentawai records show long-term trends and decadal shifts, that may in turn influence interannual variability (Fig. 6a,b) and are not adequately captured in these short coral records. Also, the development of paired δ^18^O and Sr/Ca reconstructions would be important, to compare IODE SST variability with hydrological changes in Indonesia.

Support for reduced interannual variability in the MCA comes from tropical Pacific coral δ^18^O reconstructions of ENSO variability, as well as from high-resolution sediment core data from the tropical Pacific spanning the past millennium^29,67^. These records suggest an enhanced equatorial SST gradient across the tropical Pacific comparable to a La Niña-like mean state, with colder SSTs in the central equatorial Pacific and reduced ENSO variability^29,67^ This appears to be part of a pan-tropical climatic pattern with reduced ENSO and IOD variability, enhanced equatorial SST gradients in the Pacific and Indian Ocean, and warmer mean SSTs in the West Pacific Warm Pool (Refs.^26,28,65,67^ and references therein). Consistent with this scenario, sediment cores from Indonesia show that mean temperatures in the maritime continent were warmer, while the thermocline in the eastern tropical Indian Ocean was deeper and upwelling was reduced^26,68^. A deeper thermocline in the eastern tropical Indian Ocean would explain reduced IOD variability in the MCA^15^, as seen in our MCA coral Sr/Ca record, which shows reduced seasonal and interannual SST variability in the eastern pole of the IOD, with only one potentially extreme positive IOD event, and a reduction in the skewness of the proxy data. (Note that the IOD drives SST skewness in the eastern Indian Ocean via the thermocline feedback^5^). Mean Sr/Ca data from the MCA also indicate warmer mean SSTs. However, mean SSTs inferred from coral Sr/Ca have large uncertainties associated with vital effects.

**Figure 7:**
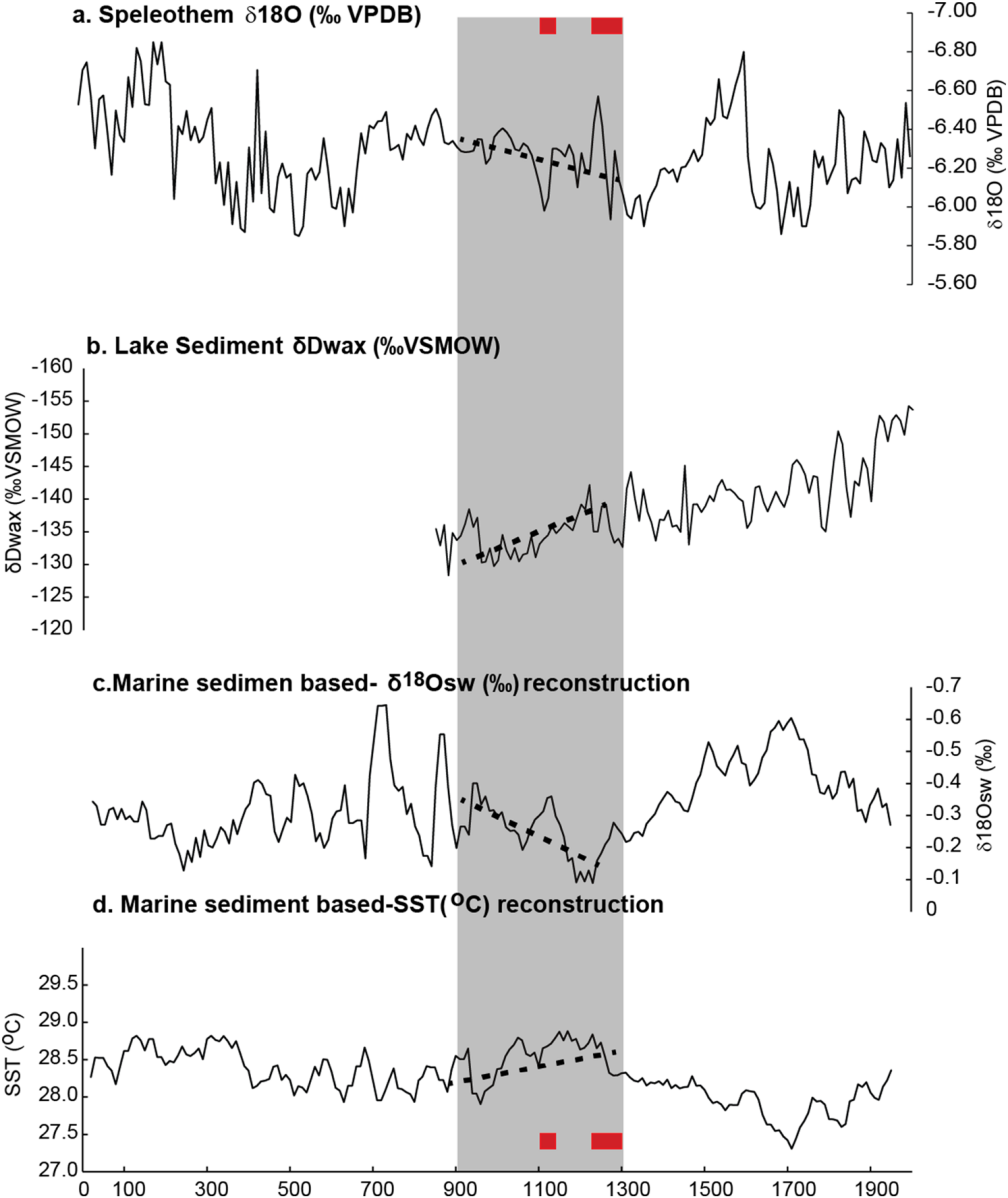
Various proxy records from Indonesia covering the MCA. (**a**) Spliced speleothem δ^18^O record (‰. VPDB)^69^, from Liang lunar cave, Flores (**b**) Lake sediment isotopic composition δDwax from Lake Lading East Java^27^. (**c**) Reconstructed δ^18^O seawater based on isotopic composition of marine sediment (foraminifers) from Makassar Strait, Sulawesi^26^. (**d**) Reconstructed SST based on Mg/Ca ratio in marine sediment from Makassar Strait, Sulawesi^26^. The data is filtered in 50 year bins^26^. Shaded grey box indicates the period of the Medieval climate anomaly (MCA). Dashed lines indicate linear trends during the MCA period. Red rectangles indicate the time period covered by high-resolution coral Sr/Ca and δ^18^O data.

We have compiled various low-resolution climate archives from various sites in Indonesia, including marine and terrestrial archives to better constrain the climatic patterns in the MCA (Fig. 7). These records suggest a heterogeneous response in the MCA, particularly with regard to hydrological changes (Fig. 7). Marine sediment cores from Makassar Strait suggest that mean SSTs were warmer than today^26,27^ with a trend towards drier conditions during the MCA^26^. (This is inferred from coupled Mg/Ca and δ^18^O measurements on foraminifera, that allows the calculation of δ^18^Osw^26^.) A drying trend during the MCA is also shown in a speleothem record from Liang Lunar Cave (Flores)^69^.

Meanwhile, the hydrogen isotopic composition of plant leaf-wax *n*-alkanes (δD_wax_) of a lake sediment core from Lake Lading in East Java shows an opposite trend towards wetter conditions. The latter would be consistent with a La Niña like/negative IOD like mean state in the MCA which should be characterized by warm SSTs and intensified convection in the West Pacific Warm Pool and in the eastern Indian Ocean^27^.

The MCA was a period when mean temperatures were warmer than today. Various low-resolution palaeoclimatological records also suggest that during the MCA, tropical climate was different from today, with a La Niña/negative IOD-like mean state with a deeper thermocline in the eastern Indian Ocean, warmer temperatures in Indonesia, and a spatially heterogenous hydrological response (e.g. Ref.^15^). At present, the western Indian Ocean warms faster than the east^60^, reducing the equatorial SST gradient in the tropical Indian Ocean and leading to an increase in IOD variability, perhaps in an unprecedented way (e.g. Refs.^28,70^). Although this may also reduce the skewness of the IOD^5^ future scenarios differ from MCA climate anomalies.

## Methods

### Analytical procedures

The modern *Porites* coral (KN2) was collected from the fringing reef of Enggano Island in approximately 5 m water depth using a pneumatic drill powered by scuba tanks. After drilling, the core was cut into 5 mm-thick slabs, X-rayed and prepared for subsampling following^61,63^. The core-top was subsampled for Sr/Ca analysis at 1 mm intervals, i.e. at monthly resolution. In this study we use the 40-year period from May 1968 to September 2007.

The sub-fossil *Porites* coral (LAM) was collected from the beach of Lampung Bay-Sunda Strait. LAM was drilled with a fuel-driven drill. Thin section analysis has been used to confirm the preservation of the coral sample and to rule out diagenetic changes^71^ (Supplementary Fig. S10).

For uranium series dating, the coral was subsampled at the GEOMAR, Helmholtz Centre for Ocean Research in Kiel, Germany. The original X-ray tomography slab (Supplementary Fig. S11) was sub-sampled at two different discrete annual growth bands, one close to the top and one closer to the bottom part of the core. In order to avoid surficial contamination fragments were cut out by a hand-hold diamond disc and reformatted rigorously down to exclusively fresh surfaces in a clean air preparation bench. These fragments were crushed to smaller fragments for binocular inspection of potential impurities. From the selected best splits of lowest pore volume 60–90 mg were dissolved in 2.25 N HNO_3_ and no remains could be observed. In brief, separation of uranium and thorium from the sample matrix was done using Eichrom-UTEVA resin following previously published procedures^72^. Determination of uranium and thorium isotope ratios was done using the multi-ion-counting inductively coupled plasma mass spectroscopy (MICICP-MS) approach on a Thermo-Scientific Neptune Plus according the method of Ref.^73^. The ages were calculated using the half-lives published by Ref.^74^. For isotope dilution measurements, a combined 233U/236U/229Th spike was used with stock solutions calibrated for concentration using NIST-SRM 3164 (U) and NIST-SRM 3159 (Th) as combi-spike, calibrated against CRM-145 uranium standard solution (formerly known as NBL-112A) for uranium isotope composition and against a secular equilibrium standard (HU-1, uranium ore solution) for the precise determination of 230Th/234U activity ratios. Whole-procedure blank values of this sample set were measured at 17 pg for thorium and 6 pg for uranium. The resulting U/Th ages are slightly different in expected succession with 0.85 ± 0.02 ky BP (bottom) and 0.81 ± 0.03 ky BP (top) which correspond to 1100–1140 ad (Supplementary Fig. S11) with present being 1950. The internal chronology of the Sr/Ca record is based on the annual growth bands, which show that LAM comprises 40 years.

The coral cores were subsampled for Sr/Ca measurements following standard procedures^37,63^. We used 0.1–0.2 mg of coral powder for Sr/Ca analysis. Sr/Ca ratios were measured at Kiel University using a Spectro Ciros CCD SOP inductively coupled plasma optical emission spectrometer (ICP-OES). Elemental emission signals were simultaneously collected and subsequently processed following a combination of techniques described by Refs.^75,76^. Average analytical precision of Sr/Ca measurements as estimated from sample replicates was typically around 0.08% RSD or less than 0.1 °C.

In this study, we compare 40-year records of monthly resolved coral Sr/Ca ratios from a modern and a sub-fossil coral from the MCA (Fig. 3).

The chronology of the modern coral Sr/Ca is developed using anchor points following the method of Ref.^37^ and the data is linearly interpolated to 12 monthly values per year. We assigned September to the Sr/Ca maxima (on average the coldest month) and May to the Sr/Ca minima (the warmest month) in any given year. We then linearly interpolated between these anchor points for all other age assignments. For correlation and linear regression analysis the Sr/Ca data from the modern core must be interpolated to monthly resolution. Similarly, the Sr/Ca data of the MCA coral from Lampung Bay has been interpolated. One year is defined manually based on the coral growth bands, and the maximum in Sr/Ca is assigned to September while the minimum is assigned to May in any given year. Basic statistics were calculated using the raw Sr/Ca data (i.e. non-interpolated Sr/Ca ratios vs. ‘depth’ in mm).

### Calibration with instrumental SST

We used SST data from the Extended Reconstructed Sea Surface Temperature version 5 (ERSST5)^49^ and OISSTv2 AVHRR^66^. Historical SST observations from the International Ocean–Atmosphere Dataset are the basis for ERSST5^49^, which has a 5°grid resolution and extends back until 1880. ERSST5 data can be compared with the modern coral Sr/Ca record over its entire time period and is routinely used to calibrate and validate coral Sr/Ca records^35,36,38,46,64^. Meanwhile OISSTv2 AVHRR^66^ is only back until 1981 November. SST data is centered at the following coordinates: 105.578 E 5.749 S (Lampung Bay, LAM) and 102.125 E 5.375 S (Enggano, KN2) (Fig. 2), i.e. the SST data is from neighbouring grid cells.

The correlation between Enggano and LAM SST is high (R = 081–0.95, N = 311, CI 95%, *p* ≤ 0.0001) and the slope is not significantly different (Supplementary Fig. S3c). Thus, it can be assumed that the SST variability at Enggano and Lampung Bay is comparable, and the modern coral Sr/Ca record from Enggano can be used as a basis to assess the MCA Sr/Ca record from Lampung Bay. Linear ordinary least squares regression of the living coral Sr/Ca record (KN2) and satellite SST (OISST, 1982–present) is used to assess the SST dependency of coral Sr/Ca: Sr/Ca = − 0.054 ± 0.003 SST + 10.45 ± 0.097 (R^2^ = 0.45 R = 0.68 N = 311, 95% CI) (Supplementary Fig. S5a). The slope of the Sr/Ca–SST relationship is consistent with published values, the negative correlation indicates that warm SST coincides with low Sr/Ca ratios in the coral skeleton^34,35,37–39,77^. We correlate also living coral Sr/Ca (KN2) with the SST coordinate LAM site, the result shows high correlation coefficient (R = 0.47–0.66, N 311, 95% CI, *p* ≤ 0.0001) (Supplementary Fig. S5c,d), this convinces us that we can use living coral at Enggano as base for working with dead coral record from LAM site because the SST variability of both Enggano and LAM site reflect similar condition.

Monthly climate indices used in this study are taken from the KNMI climate explorer (https://climexp.knmi.nl/). We use the Niño 3.4 index^78^ (based on NOAA ERSSTv5, ONI), the dipole mode index (DMI)^1^ and the south east equatorial Indian Ocean SST anomaly (SEIO) index, which captures SST variability in the eastern pole of the IOD (all based on ERSSTv5). These indices characterize the dominant climatic modes that impact the study area: Niño 3.4 captures ENSO-related SST variability in the equatorial Pacific, the DMI index the east–west SST gradient in the equatorial Indian Ocean, and the SEIO index SST variability in eastern pole of the IOD (referred to as IODE in this paper). 3 months running mean data of SST and coral Sr/Ca are used for a linear OLS regression between SST (coral Sr/Ca) and the climate indices (i.e. Niño 3.4, DMI and IODE). IOD events are selected based on the list of positive and negative IOD years from the Australian Government Bureau of Meteorology (http://www.bom.gov.au/climate/iod/). A positive (negative) IOD event occurs when the DMI index is > 0.4 °C (< − 0.4 °C). El Niño and La Niña events are selected from the list provided by the National Oceanic and Atmospheric Administration (NOAA) climate prediction centre-Ocean Niño Index (ONI) (https://origin.cpc.ncep.noaa.gov/products/analysis_monitoring/ensostuff/ONI_v5.php). A 0.5 °C threshold of ONI is defined as El Niño/La Niña events.

### Power spectrum analysis

The power spectrum analysis^79^ is applied on both modern and subfossil coral Sr/Ca (Supplementary Fig. S9). The rectangle window is applied in the annual variability of coral Sr/Ca with 5 segments and 2 oversamples, significance level 95%. In sub-fossil coral, the significant high-power spectrum is shown at 0.015 frequency band which is represent 66.7 year/cycle for confidential interval of 95% and in modern coral significant power spectrum is shown at 0.32 frequency band equal with 3.2 year/cycle.

## Supplementary Information

Supplementary Figures.
