# Supplementary material for: Modern and sub-fossil corals suggest reduced temperature variability in the eastern pole of the Indian Ocean Dipole during the medieval climate anomaly
Source: Sci Rep. 2021 Jul 22;11:14952. doi: 10.1038/s41598-021-94465-1 (PMC8298714; doi:10.1038/s41598-021-94465-1)
Supplement: Supplementary file 1 — Supplementary Figures. [file 41598_2021_94465_MOESM1_ESM.pdf]

SUPPLEMENT INFORMATION

This is supplement information for article entitled:

Modern and sub-fossil corals suggest reduced temperature variability in the eastern pole of the Indian Ocean Dipole during the medieval climate anomaly

Sri Yudawati Cahyarini[1,2,3*], Miriam Pfeiffer[2,3], Lars Reuning[2,3], Volker Liebetrau[4], Wolf-Chr. Dullo[4], Hideko Takayanagi[5], Iwan P. Anwar[6], Dwi A. Utami[1], Dieter G. Schönberg[3], Marfasran Henrizan[1], Anton Eisenhauer[4]

1. Paleoclimate & Paleoenvironment Research Group, Res. Cent. for Geotechnology-Indonesian Institute of Sciences (LIPI), Bandung, 40135, Indonesia
2. RWTH Aachen University, Geology and Paleontology, Aachen, 52056, Germany
3. Kiel University, Institute of Geosciences, Kiel, 24118, Germany
4. GEOMAR Helmholz Centre for Ocean Research, Kiel, 24148, Germany
5. Institute of Geology & Paleontology, Graduate School of Science, Tohoku University, Sendai, Japan
6. Institute Teknologi Bandung, Dept. Oceanography, Bandung,40132, Indonesia

Corresponding author * Sri Yudawati Cahyarini, address: Indonesian Institute of Sciences (LIPI)-R.C. for Geotechnology, Komplek LIPI Gd. 70-80 Jl Sangkuriang Bandung-40135, Indonesia. Email: sycahyarini@gmail.com; sriy004@lipi.go.id

The supplement consists of 11 Figures :

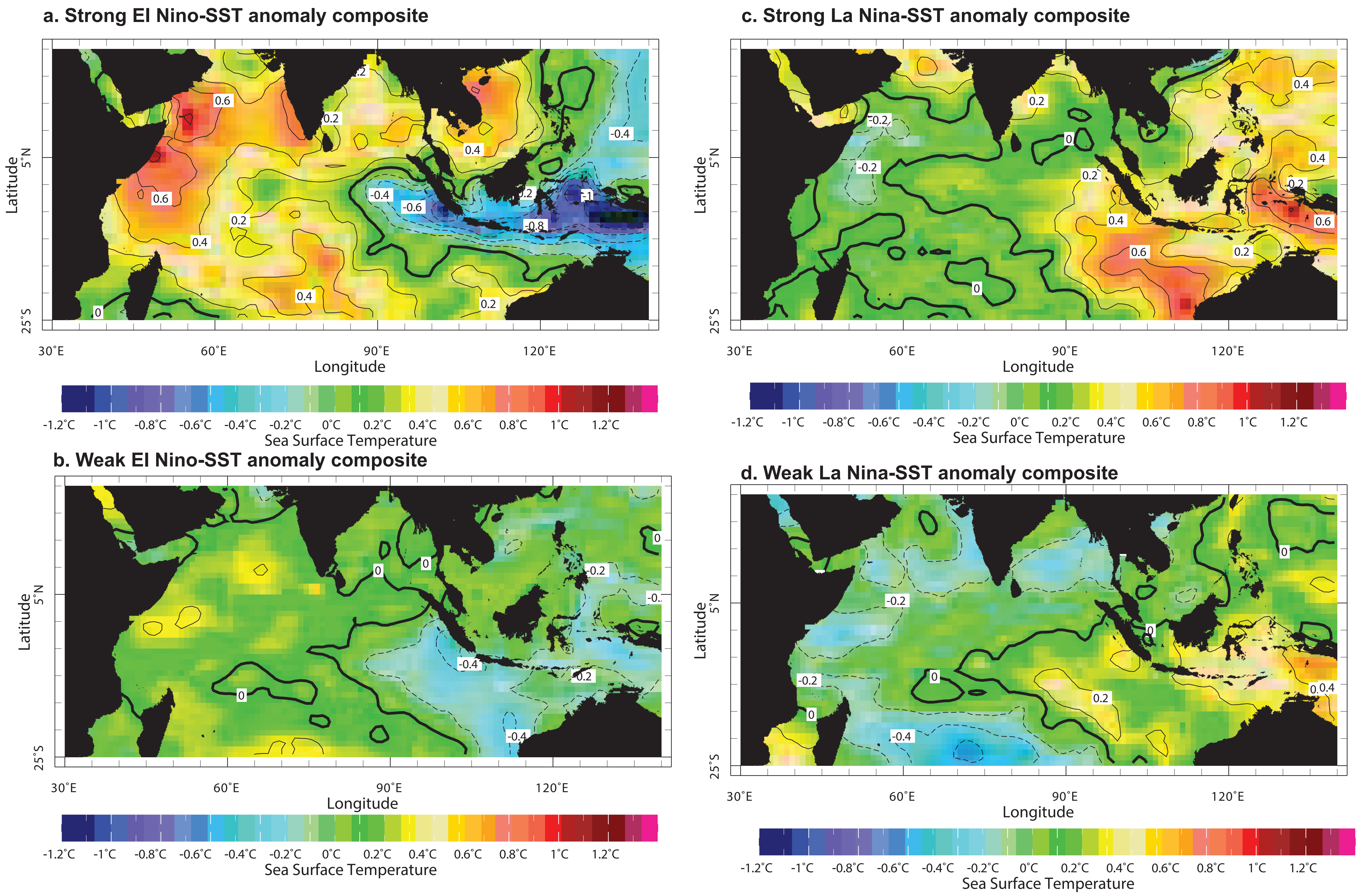

Figure S1. Composite maps of ENSO events. (a,b) Composite SST anomalies during strong and weak El Niño and (c,d) strong and weak La Niña events. SST data is taken from the OISST v2 dataset. Composite SST anomaly data is averaged over September-November season during each event. ENSO year events are clasified based on the National Oceanic and Atmospheric Administration (NOAA) climate prediction centre-Ocean Niño Index (ONI) (https://origin.cpc.ncep.noaa.gov/products/analysis_monitoring/ensostuff/ONI_v5.php). Map is generated using expert mode menu in https://iridl.ldeo.columbia.edu/SOURCES/.NOAA/.NCDC/.OISST/.version2/.AVHRR/.sst/

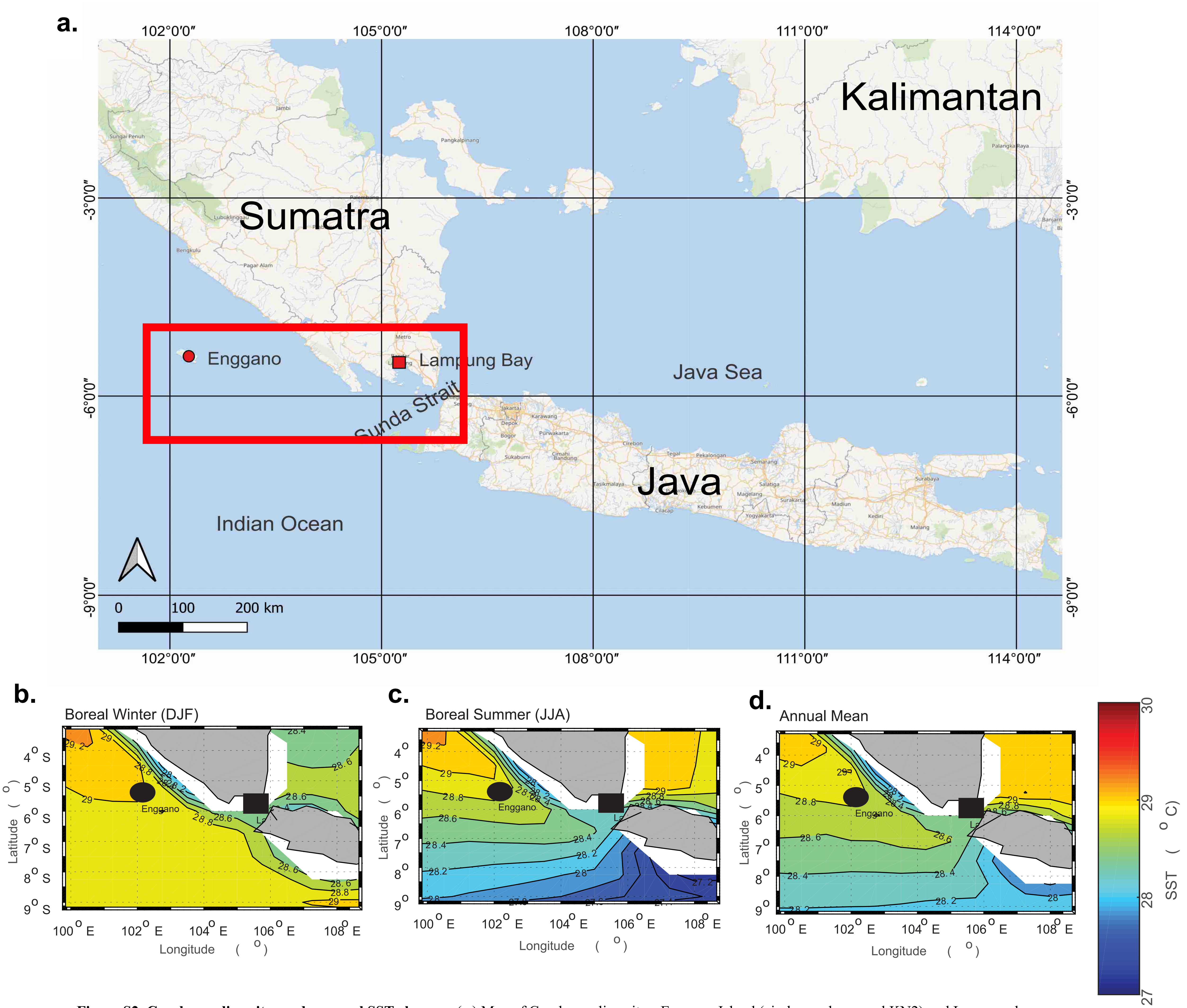

Figure S2. Coral sampling sites and seasonal SST changes. (a.) Map of Coral sampling sites: Enggano Island (circle, modern coral KN2) and Lampung bay, Sunda Strait (rectangle, LAM, MCA coral). Map of sea surface temperatures (SSTs) during (b.) boreal winter, (c.) boreal summer and (d.) annual means. Map (a.) is generated from Wikimedia Maps in OpenLayers plugin QGIS ver. 3.14.1-Pi and Map (b-d) is generated using the MATLAB version 2017 with online script version 1.4m available at www.eoas.ubc.ca/~rich/map.html [80]. SST data is obtained from ERA-Interim data source (https://www.ecmwf.int/en/forecasts/datasets/reanalysis-datasets/era-interim).

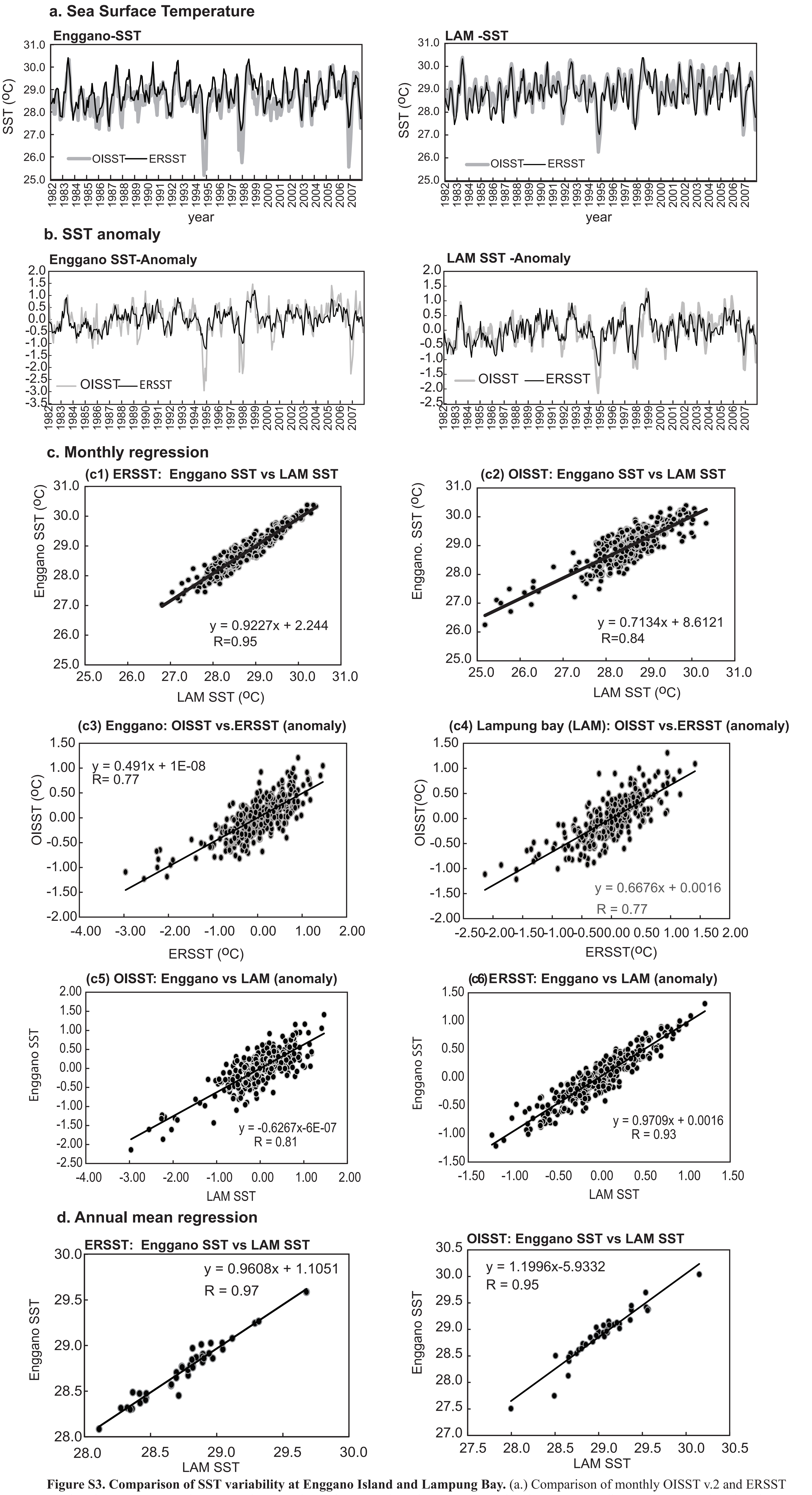

Figure S3. Comparison of SST variability at Enggano Island and Lampung Bay. (a.) Comparison of monthly OISST v.2 and ERSST v.5 data from Enggano and Lampung bay, Sunda Strait (LAM site). (b.) Same as (a.) but for SST anomalies (mean seasonal cycle subtracted). (c.) Linear regression of (c:1,2) monthly Enggano SST and LAM SST from ERSST (left) and OISST (right). (c:3,4.) Scatter plots and linear regression of OISST versus ERSST, monthly anomalies, for Enggano Island and Lampung bay, Sunda strait. OISST contains satellite data and is available since Nov 1981. Note that satellite SST shows a stronger cooling during the extreme positive IOD events of 1994, 1997 and 2006. Correlation between SST data for the grids including Enggano and LAM using OISST (c5.) and ERSST (c6.). (d.) Annual mean regression of Enggano and LAM SST: ERSST (left) and OISST (right).

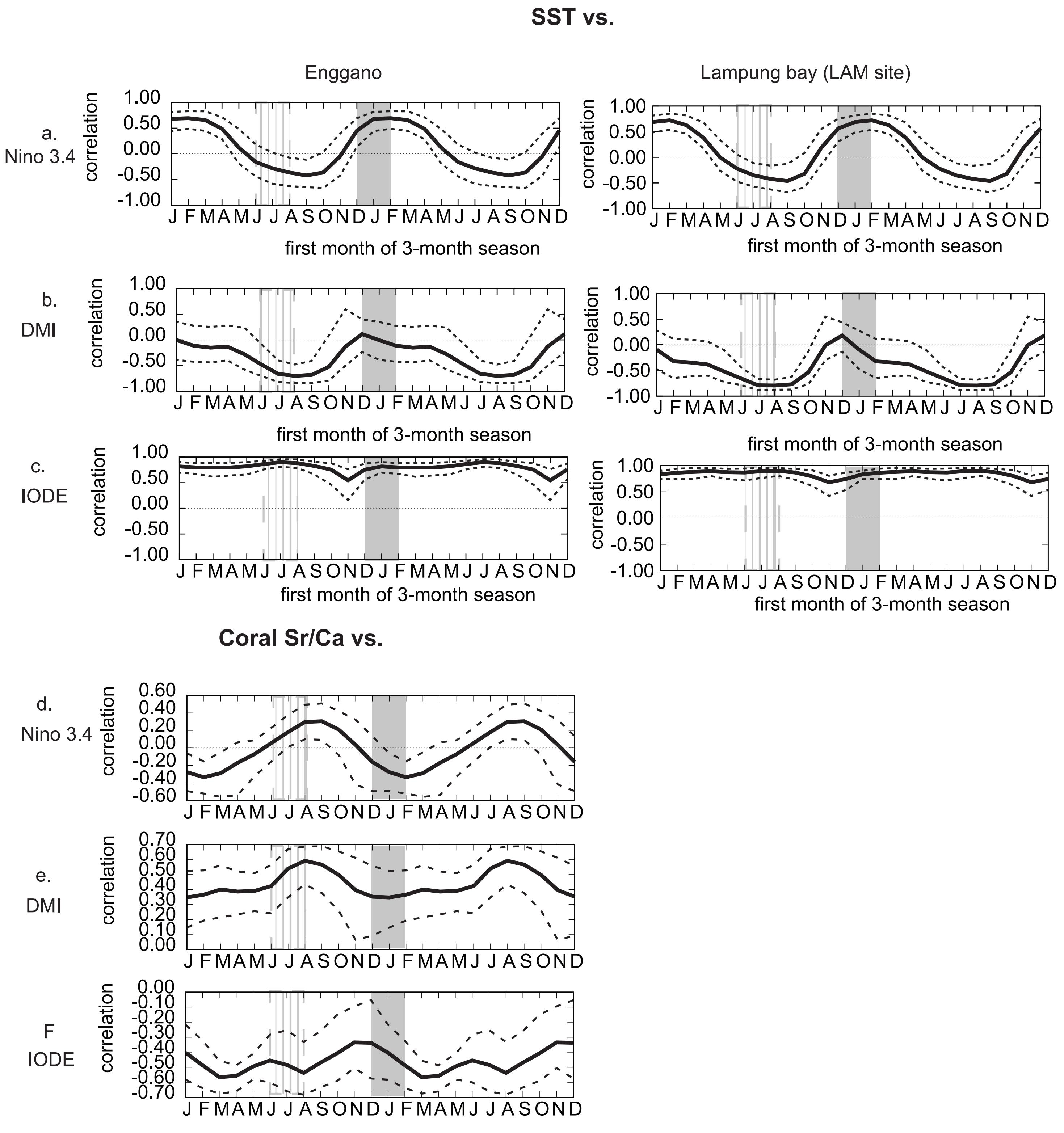

Figure S4. Seasonal correlation of Enggano and Lampung Bay SST with ENSO, the IOD index and IODE. (a, b, c) Correlation of satellite SST (OISST) from the grids including Enggano Island and Lampung Bay with (a.) the Nino 3.4 index, (b.) the DMI index, (c.) IODE SST. (d., e., f.) Correlation of the modern coral Sr/Ca record from Enggano Island with (d.) the Nino 3.4 index, (e.) the DMI index, and (f.) IODE SST. Box with grey lines: South East monsoon season, grey box: North West monsoon season.

Monthly Calibration

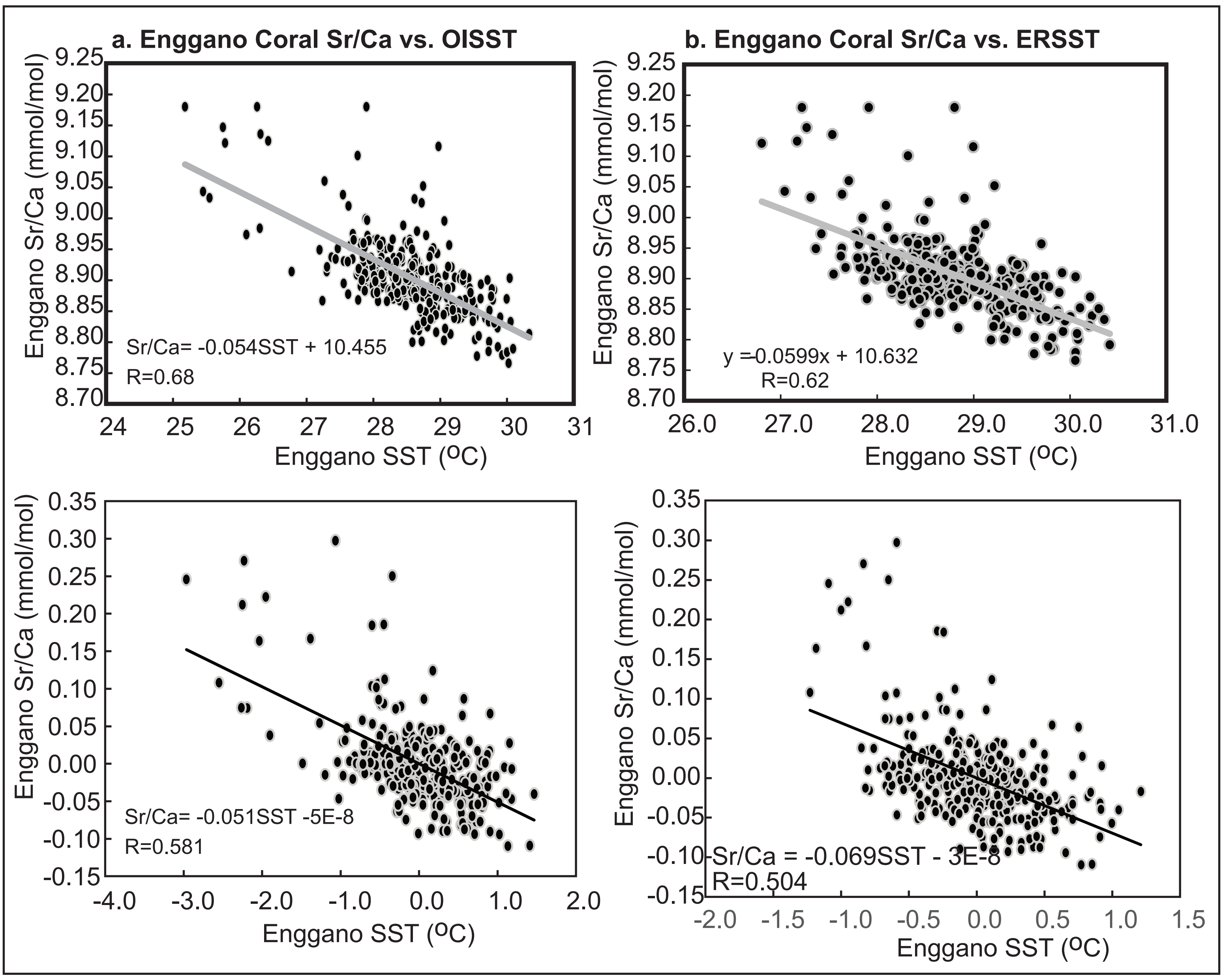

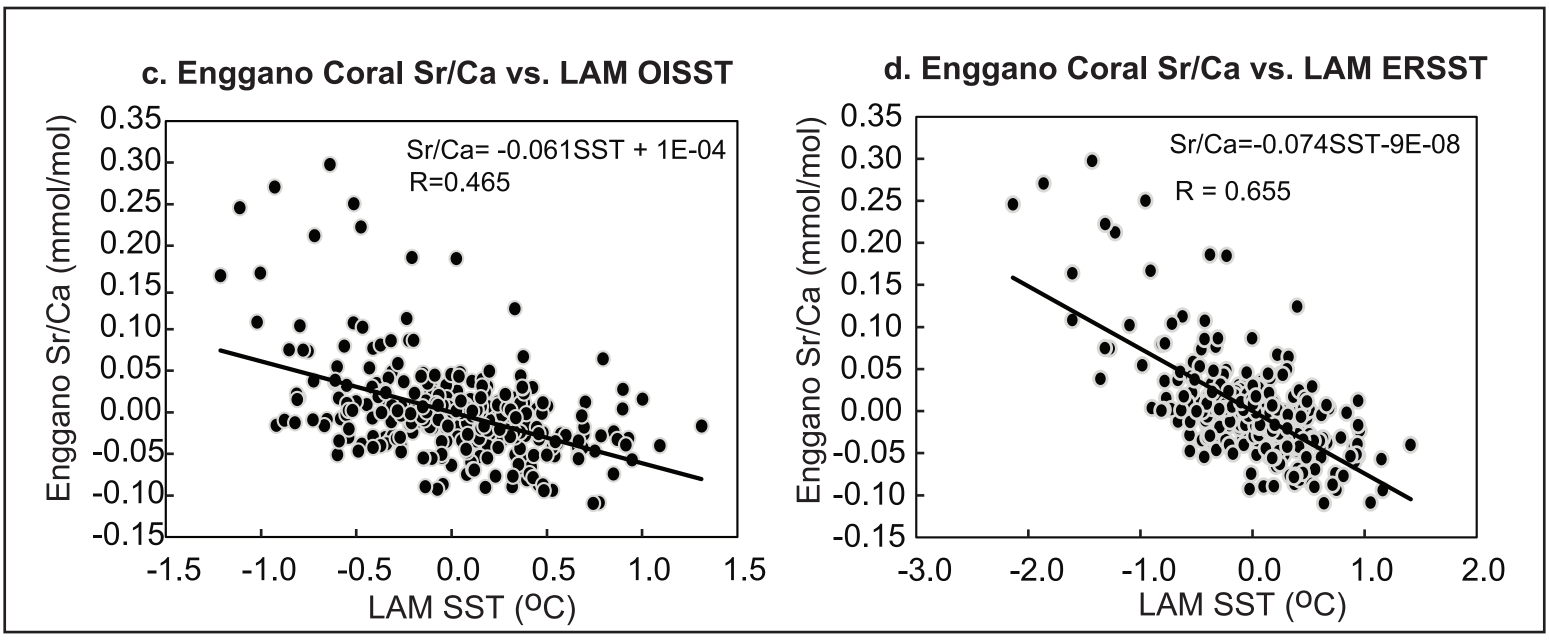

Annual mean Calibration

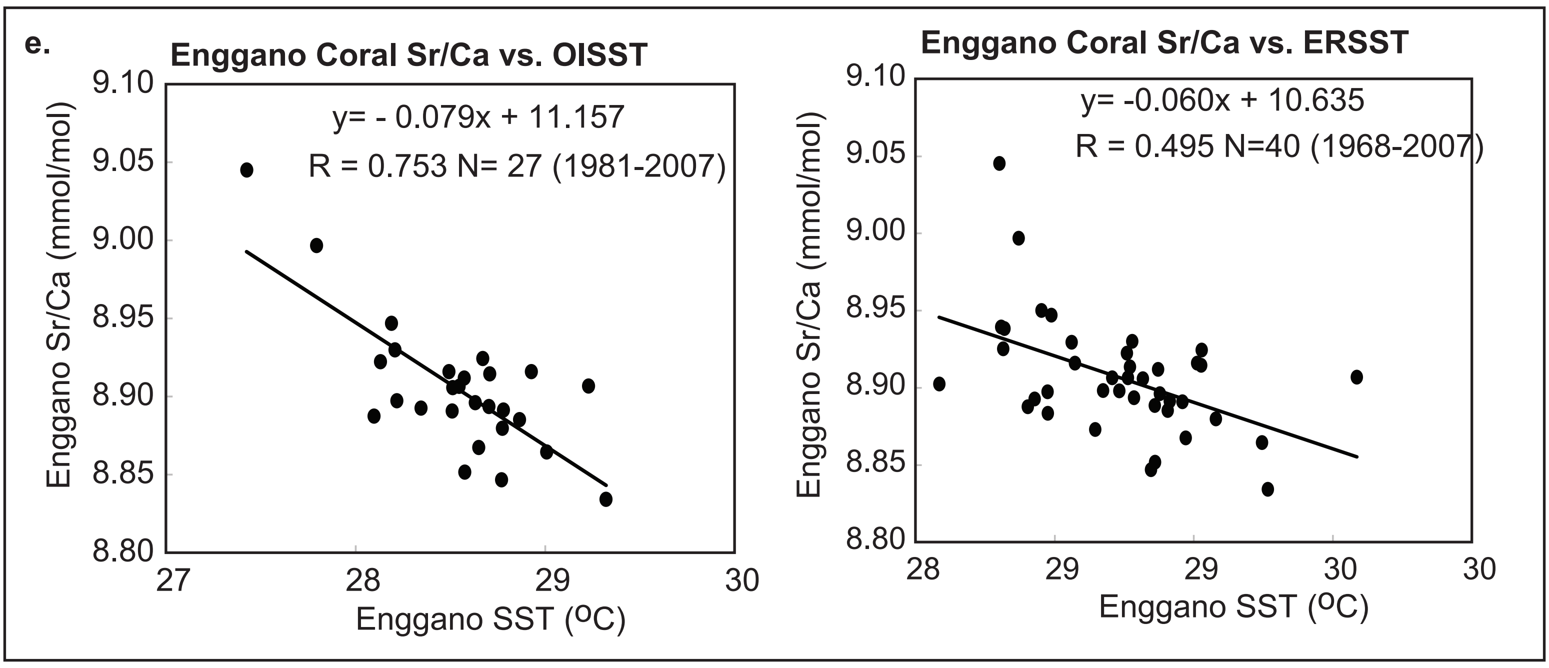

Figure S5. Calibration of monthly coral Sr/Ca record from Enggano Island. (a.) Linear, ordinary least squares regression of OISST v2. and coral Sr/Ca data from Enggano Island (KN2) for the time period from November 1981 to September 2007 resulted in a significant correlation (n=311, R=0.68-0.58, p ≤0.0001, ci. 95%) (upper panel: monthly data, bottom panel: monthly anomalies). (b.) Same as A. using ERSST v5, similarly resulted in a significant correlation (n=311, R=0.62-0.50, p ≤0.0001, ci. 95%) (upper panel: monthly data, bottom panel: monthly anomalies). (c., d.) Correlation of the Enggano coral Sr/Ca record (monthly anomalies) with SST anomalies from the coordinate including Lampung Bay using: (c.) OISST and (d.) ERSST. This shows that the Enggano coral Sr/Ca records SST in a wider area, including Lampung Bay. (e.) Annual mean calibration of coral Sr/Ca from Enggano Island with SST from OISST (left panel) and ERSST (right panel).

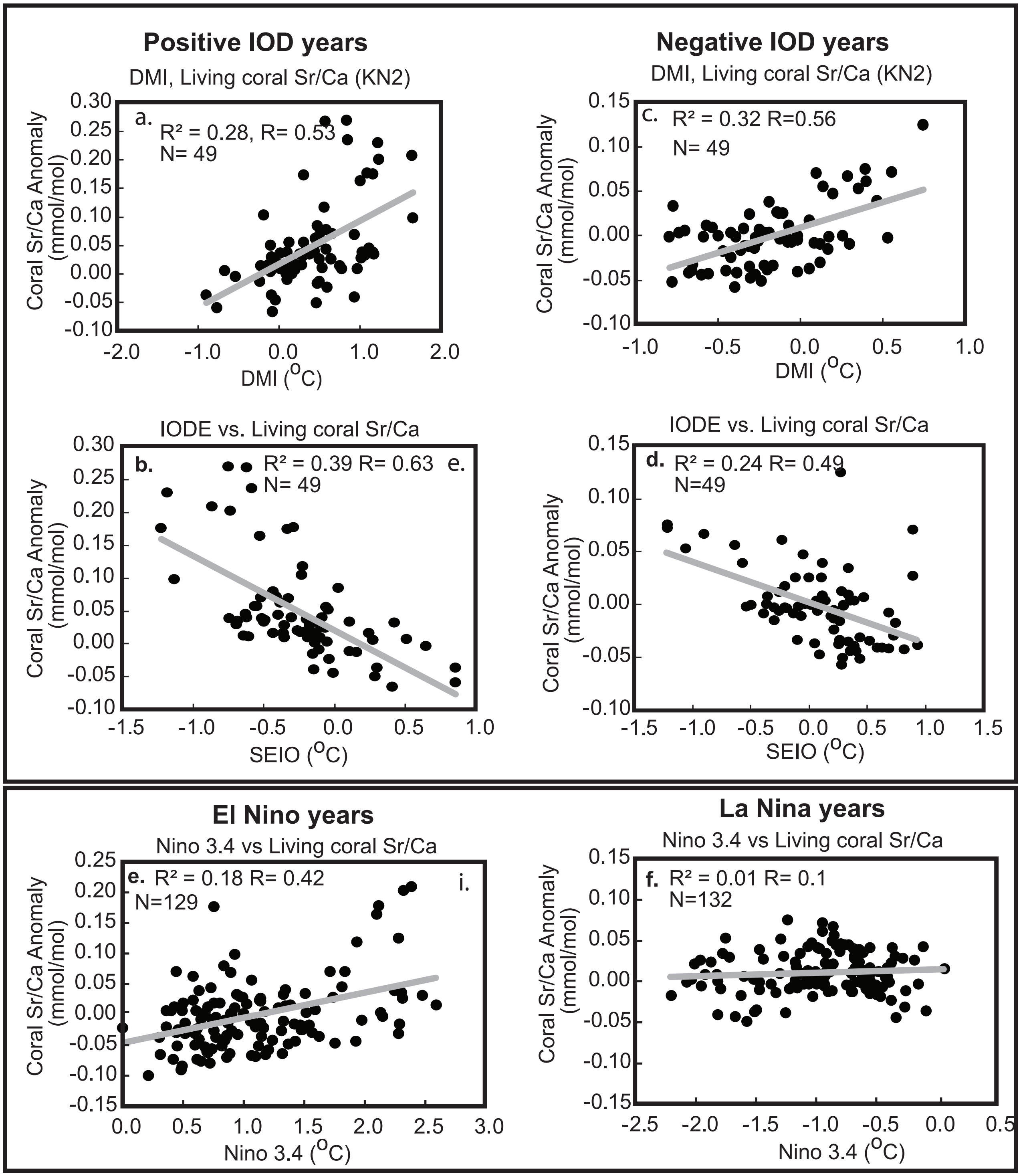

Figure S6. (a-d) Scatter plots of positive (left) and negative (right) IOD events as recorded in the DMI (top) and IODE (bottom) SST index vs. modern coral Sr/Ca data from Enggano Island. (e-f) Scatter plots El Nino (left) and La Nina (right) events as recorded in the Nino 3.4 index vs. modern coral Sr/Ca data from Enggano Island. IOD events are selected according to the event list of the Australian Bureau of Meteorology (http://www.bom.gov.au/) (IOD) and ENSO events from NOAAs Climate Prediction Centre (https://origin.cpc.ncep.noaa.gov). All monthly values exceeding the threshold for IOD (+/- 0.4°C) and ENSO (+/- 0.5°C) events are selected for this regression (see text for discusssion).

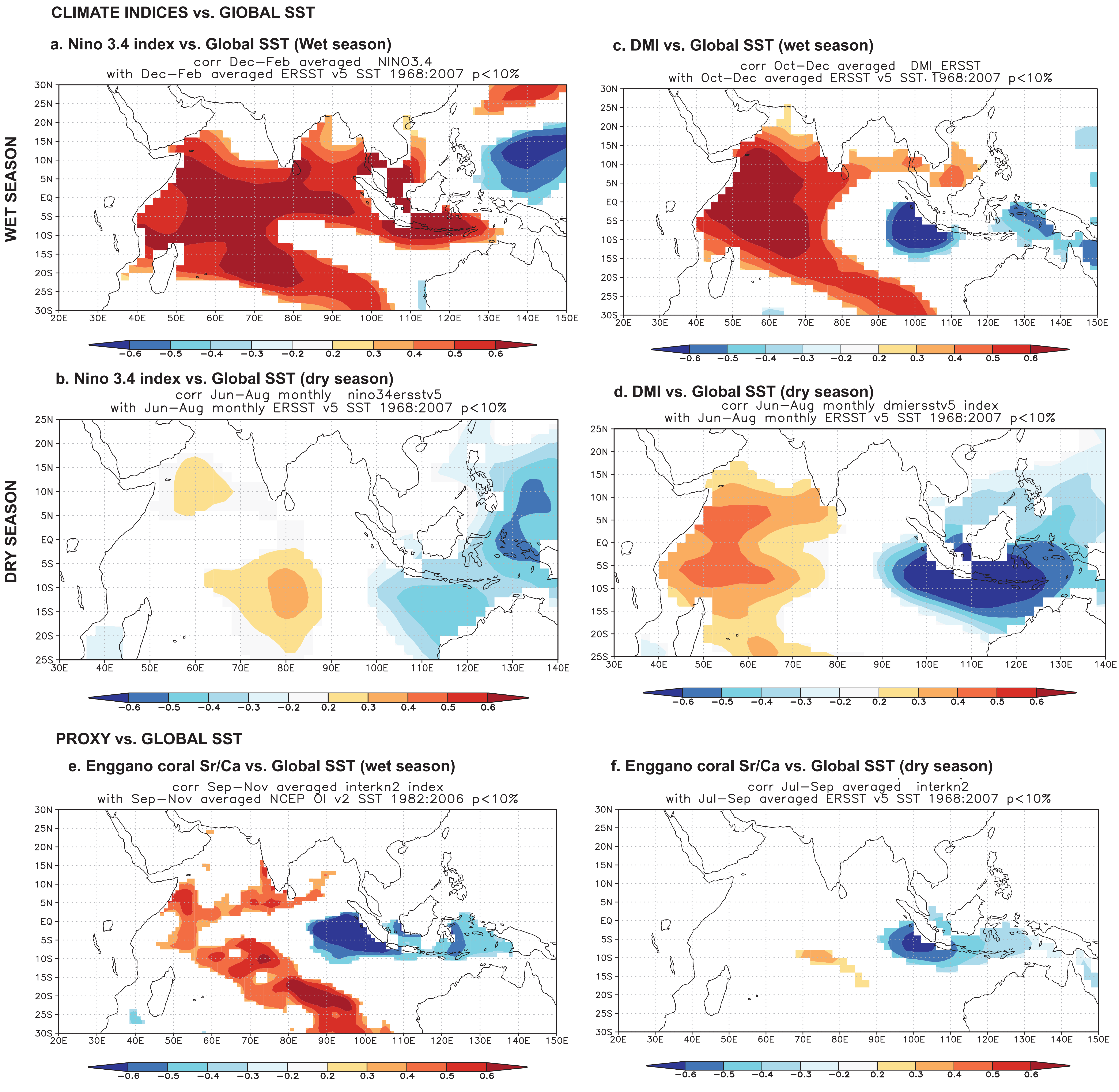

Figure S7. Spatial correlation of ENSO and the IOD in the Indian Ocean during the wet and dry season in Indonesia. (a., b.) Nino 3.4 index and SST, (c., d.) DMI and SST. SST data is from the ERSST v5 dataset. The correlation between Nino 3.4 and SST is lower during boreal summer and winter than the correlation between the DMI and SST in the South eastern Indian Ocean. This shows that ENSO-related SST changes in the South eastern Indian Ocean are weaker than the IOD-related SST changes. The field correlation between the Nino 3.4 index and SST shows low correlations in South Eastern Indian Ocean that range between $0.4<R<0.5$ during the onset of ENSO, which is usually develops in September-November. This usually corresponds to the wet season in Indonesia. (e., f.) Field correlation of coral Sr/Ca from Enggano (KN2) with global SST during (e.) the wet season and (f.) the dry season of Indonesia. Note the strong negative correlation (reflecting the negative Sr/Ca-SST relationship) in the South-eastern Indian Ocean, corresponding to the eastern pole of the IOD (and including Lampung Bay). Map is generated using KNMI explorer: https://climexp.knmi.nl/start.cgi, correlate with a field, averaging over 3 months.

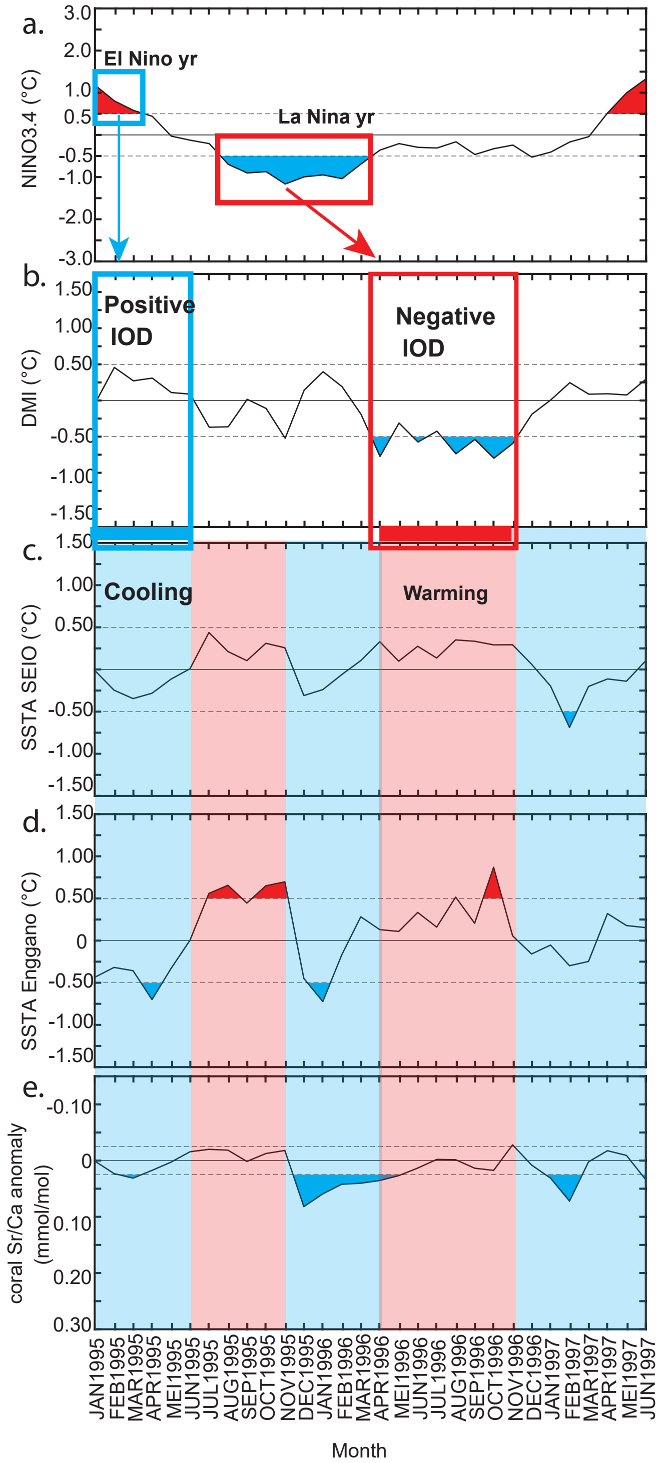

Figure S8. January 1995 to July 1997 time series of (a.) the Nino 3.4 index (b.) the DMI, (c.) IODE SST, and (d.) monthly SST anomalies in the grid including Enggano Island (OISST v2). (e.) Coral Sr/Ca anomalies from the modern Enggano Island coral (KN2). Blue shading indicates cooling, red shading warming. Blue (red) boxes in (a.) and (b.) indicate the occurrence of an El Nino/positive IOD (La Nina/negative IOD) event (see text for discussion).

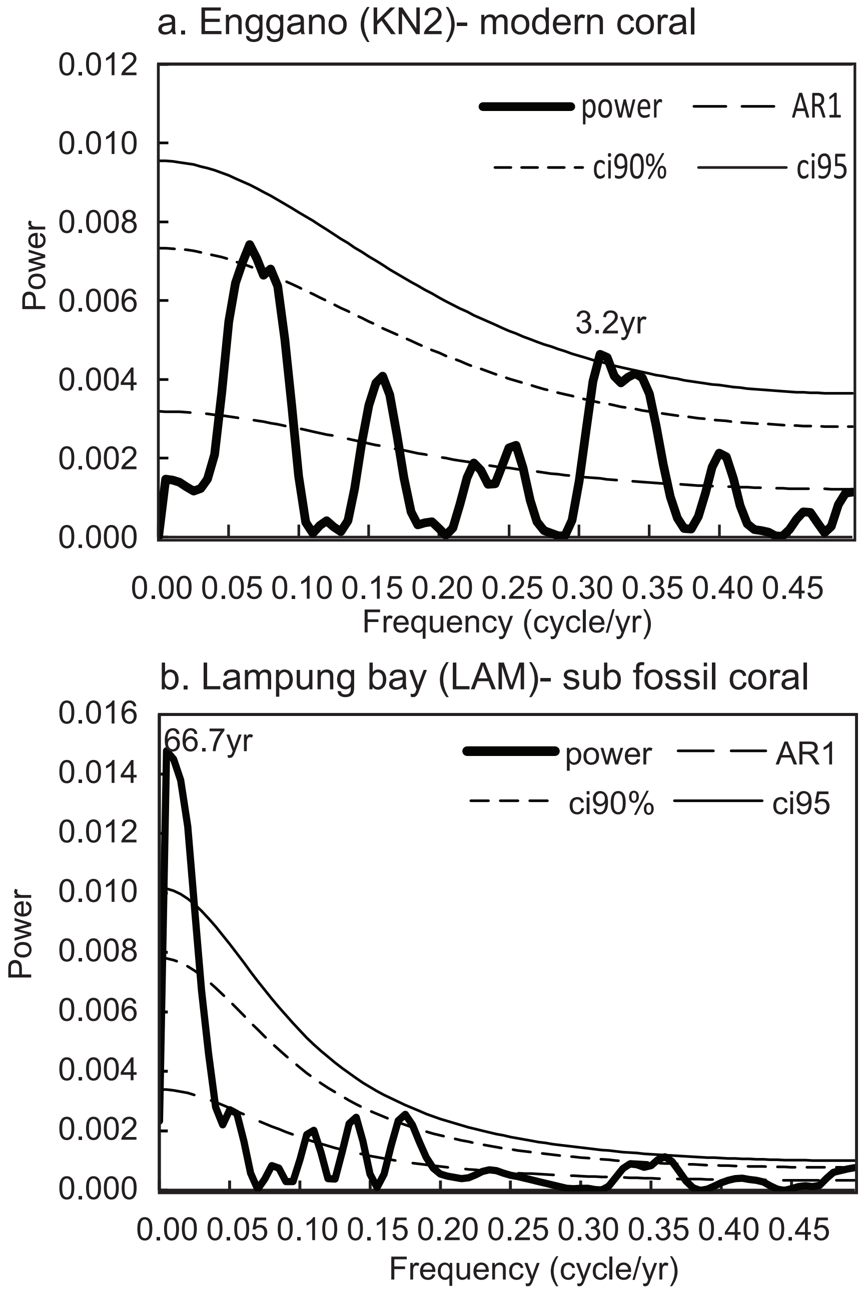

Figure S9. Power spectrum of (a.) modern coral Sr/Ca from Enggano and (b.) sub-fossil, MCA coral LAM. Modern coral shows significant power at interannual periods (3.2 yrs), significant at the 95% CI. Meanwhile, the MCA coral shows a long-term trend (~66.7yrs, exceeding the length of the coral record), but lacks significant interannual variability.

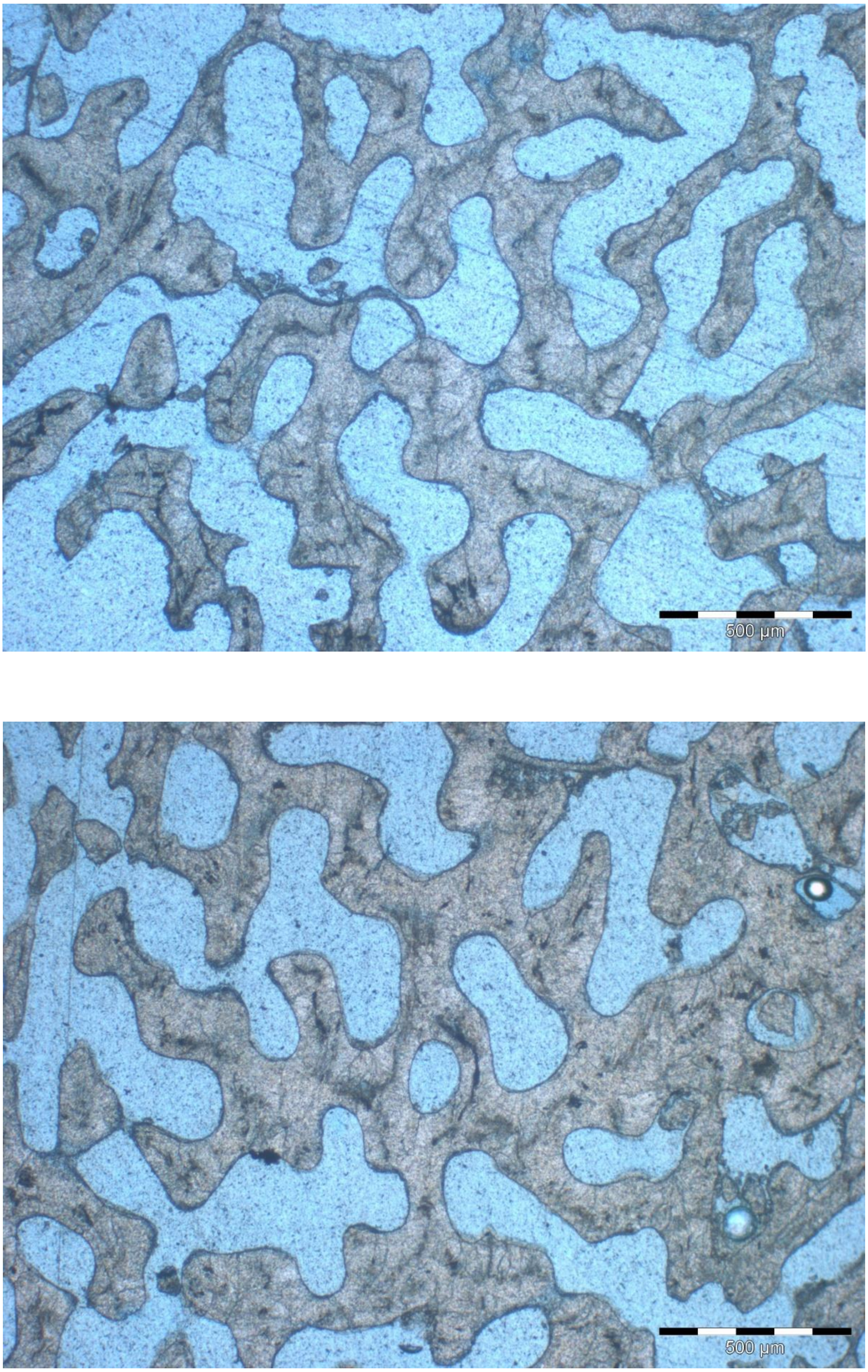

Figure S10. Thin section photograph of the MCA coral from Lampung Bay shows a well-preserved coral skeleton.

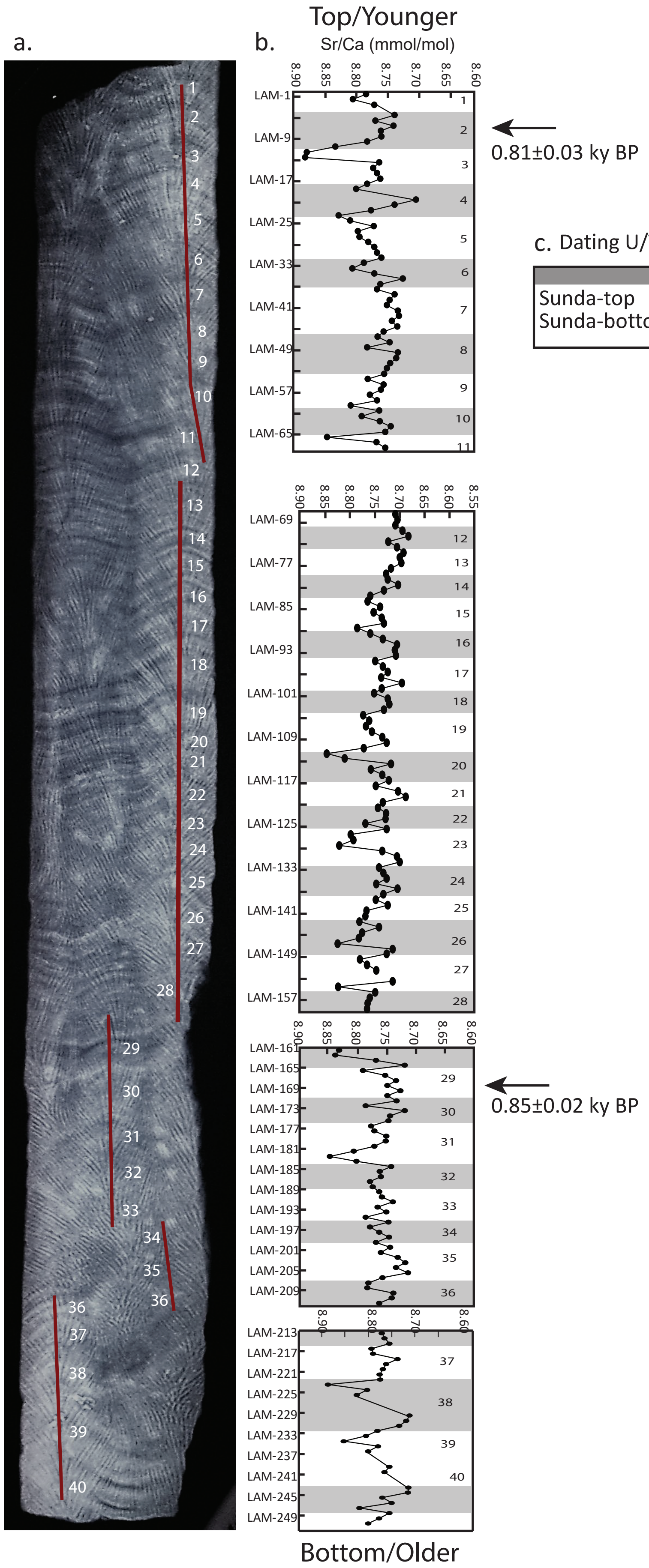

c. Dating U/Th

	ka	±		δ 234 U	±
Sunda-top	0,81	0,03	2,2%	148,1	3,6
Sunda-bottom	0,85	0,02	1,6%	146,6	4,1

Figure S11. (a.) Coral X-radiograph showing annual growth bands and the sampling transects (red line). The number of years derived from the counting of annual bands (high/low density band couplets) is given (white numbers). (b.) Measured coral Sr/Ca ratios along the sampling transects. Annual growth bands derived from the x-ray image are indicated (shading and numbers). Arrows indicate the location of the U/Th samples. (c.) Table with results from U/Th dating.
